# Differential Glioma-Associated Tumor Antigen Expression Profiles of Human Glioma Cells Grown in Hypoxia

**DOI:** 10.1371/journal.pone.0042661

**Published:** 2012-09-05

**Authors:** Lisheng Ge, Andrew N. Cornforth, Neil T. Hoa, Christina Delgado, Shiun Kwei Chiou, Yi Hong Zhou, Martin R. Jadus

**Affiliations:** 1 Diagnostic and Molecular Health Care Group, Veterans Affairs Medical Center, Long Beach, California, United States of America; 2 California Stem Cell Inc., Irvine, California, United States of America; 3 Research Service Healthcare Group, Veterans Affairs Medical Center, Long Beach, California, United States of America; 4 Neuro-Oncology Program, Chao Comprehensive Cancer Center, University of California Irvine, Orange, California, United States of America; 5 Department of Neurological Surgery, University of California Irvine, Irvine, California, United States of America; 6 Department of Biological Chemistry, University of California Irvine, Irvine, California, United States of America; 7 Pathology and Laboratory Medicine, University of California Irvine, Irvine, California, United States of America; City of Hope National Medical Center and Beckman Research Institute, United States of America

## Abstract

Human U251 and D54 glioma cells were tested for expression of 25 glioma-associated tumor antigen precursor proteins (TAPP) under hypoxic (1% O_2_) or normoxic (21% O_2_) conditions. Hypoxic glioma cell lines increased their mRNA expression for nine TAPP (Aim2, Art-4, EphA2, EZH2, Fosl1, PTH-rP, Sox 11, Whsc2 and YKL-40), as assessed by quantitative reverse transcriptase real-time/polymerase chain reaction (qRT-PCR). Increased differences with three hypoxic-induced TAPP: EZH2, Whsc2 and YKL-40 were shown at the protein levels by fluorescent antibody staining and quantitative electrophoretic analysis. Two TAPP (MRP3 and Trp1) were down-regulated by hypoxia in glioma cell lines. Growing the glioma cells under hypoxia for 13 days, followed by returning them back to normoxic conditions for 7 days, and restored the original normoxic TAPP profile. Thus, hypoxia was an environmental factor that stimulated the transient expression of these antigens. Intracranial xenografts grown in nude mice derived from U251 cells that had been cultured under neurosphere stem cell conditions showed increased expression of Whsc2 or YKL-40, demonstrating that these *in vitro* properties of glioma also occur *in vivo*. Whsc2-specific cytotoxic T lymphocytes killed the hypoxic U251 glioma cells better than normoxic glioma cells. The antigens expressed by hypoxic tumor cells may be a better source of starting tumor material for loading dendritic cells for novel immunotherapy of glioma using tumor-associated antigens.

## Introduction

Normal atmosphere contains 21% oxygen (O_2_) (150 mm Hg is defined as normoxic). Freshly oxygenated blood leaving the lungs contains about 13% O_2_, while the blood returning to the lungs possesses about 5% O_2_ [1]–[4]. Blood entering the brain shows the same range of oxygen concentrations [5]–[7]. Due to irregular cellular vascularization in any cancer, there are various oxygen gradients within any tumor. Hypoxic conditions are usually defined as less than 5% O_2_; severe hypoxia or anoxic (0.02% O_2_) regions occur deep in central cores of tumors. The lack of oxygen regulates many genes and helps explain differences in tumor behavior reviewed in [8]–[11]. Extreme hypoxia kills glioma cells and helps explain the pseudo-palisading necrosis that is a hallmark characteristic of Glioblastoma Multiforme (GBM) [10]. More pronounced hypoxia in the glioma correlates with poorer prognosis [9], [12]. Hypoxia can also alter immune responses, but can open up treatment opportunities [13]–[15]. Experimentally, it is concluded that oxygen tension between 0.5% and 2.5% is considered to be the best model of glioma hypoxia [8].

Immunotherapy as an adjunct therapy [16]–[24] is beginning to improve outcomes for some glioma patients. Currently, the most-widespread application of immunotherapy towards GBM uses *ex vivo* activated DC [18]–[24]. Here the patient's peripheral monocytes are stimulated with interleukin-4 (IL-4) and granulocyte-macrophage colony stimulating factor (GM-CSF). These immature DC are then pulsed with tumor-associated antigens, which can include killed intact tumor cells, antigen-encoding RNA, soluble tumor homogenates or synthetic peptides [25] [Bibr pone.0042661-Toda1] [Bibr pone.0042661-Heimberger1] [Bibr pone.0042661-Izumoto1] [Bibr pone.0042661-Parney1]–[30]. The antigen-pulsed DCs are administered as a therapeutic vaccine, which in turn stimulates anti-tumor T cell immunity.

Resected tumors are used for pathological and molecular analysis, and various diagnostic tests. The remaining tumor sections after these samplings are made are then used for immunotherapy. Hence the vaccinating tumor material could come from either well-oxygenated regions of the tumor or hypoxic/necrotic regions, depending upon how that tissue was processed on that given day. As long as tumor cells are present, it is assumed that this cancer antigen expression is representative of the tumor and it should suffice for proper dendritic cell loading. Jarboe, *et al.*, [31] showed that in glioma tumors, there are variable regions of IL-13Rα2 expression. Likewise, Little and colleagues [32] showed heterogeneity of human gliomas with respect to various tyrosine kinase-based receptors. Human glial tumors express a wide variety of tumor-associated antigens (TAA) and could be theoretically considered very antigenic [33], [34]. No one has ever shown that one region of a tumor produces superior DC-T cell stimulation than any other region of the tumor. In this paper, we investigate whether hypoxia can alter some glioma-associated antigen selection that could have important ramifications for DC-based immunotherapy.

We characterized the antigenic profiles of two well-known human glioma cell lines, U251 and D54. We used these cell lines since they are well-established (over 30 yrs old) and are relatively homogenous and stable. We evaluated twenty five known glioma-associated antigenic precursor proteins (TAPP) and their mRNA level as detected by using quantitative real time polymerase chain reaction (qRT-PCR). These antigens were examined since they were reported to be made by human gliomas and human T cell immune responses are documented against those antigens. Table S1 highlights the salient findings of each of these antigens and their relevance towards generating immune responses against human brain tumors.

Human glioma cells were grown for a week under standard incubator conditions (21% O_2_) or under conditions that represent typical hypoxia (1% O_2_). Nine TAPPs from the glioma cells (Aim2, Art-4, EphA2, EZH2, Fosl1, PTH-rP, Sox11, Whsc2 and YKL-40) consistently exhibited increased mRNA presence (≥1.9-fold expression) after culturing the cells in hypoxia (1% O_2_). Two other antigens (MRP3 and Trp-1) showed consistent decreases in response to hypoxia (≥−1.9 fold reduction relative to normoxic conditions). The three most hypoxia-induced tumor antigen precursor proteins (EZH2, Whsc2 and YKL-40) were confirmed to be up-regulated at the protein level by doing immunofluorescent microscopy and intracellular flow cytometry with the glioma cell lines. The altered TAPP expression in cells grown under hypoxia for 13 days reverted back to the normoxic baseline values when the glioma cells were returned to normoxic conditions for one week. This experiment indicates that these hypoxic-specific responses are reversible. The data shown here indicate that some tumor antigen profiles do have an oxygen dependent characteristic to them. Human CTLs specific for Whsc2 demonstrated enhanced killing on the hypoxic U251, indicating that the increased hypoxic expression may have biological significance. These results suggest that the initial source of antigenic material can effect generating effective immune responses.

## Results

### Tumor antigen precursor protein (TAPP) differences between the normoxic and hypoxic cells

Under normoxic conditions, one million U251 and D54 cells were placed into 15 mls of media within a T75 cm^2^ flask and were cultured under the two different conditions (normoxia or 1% O_2_ hypoxia). No morphological changes were empirically observed with any cells when the U251 and D54 cells were grown under normoxic or 1% O_2_ (hypoxic) conditions. The growth of both cell lines was much slower when cultured under hypoxic conditions when compared to standard normoxic (21% O_2_) conditions. After 1 week of culture both the U251 and D54 cells were confluent when grown under normoxic conditions. While under hypoxic conditions for 1 week, the U251 cells reached 60–70% confluence, while the D54 cells achieved a 40–50% confluence. Cells counted by a hematocytometer confirmed this altered growth response (data not shown). These different proliferative rates probably reflect the different metabolic pathways being used by these cells to meet their energy requirements. The U251 cells are reported to use glycolytic and oxidative phosphorylation pathways, while D54 cells only use oxidative phosphorylation processes for their energy requirements [35], [36]. The yield of total RNA of the hypoxic cells was reduced, most likely due to the fewer cells being present at the time of harvesting. No problems were seen when the RNA was isolated and converted into cDNA from the hypoxic cells.

**Figure 1:**
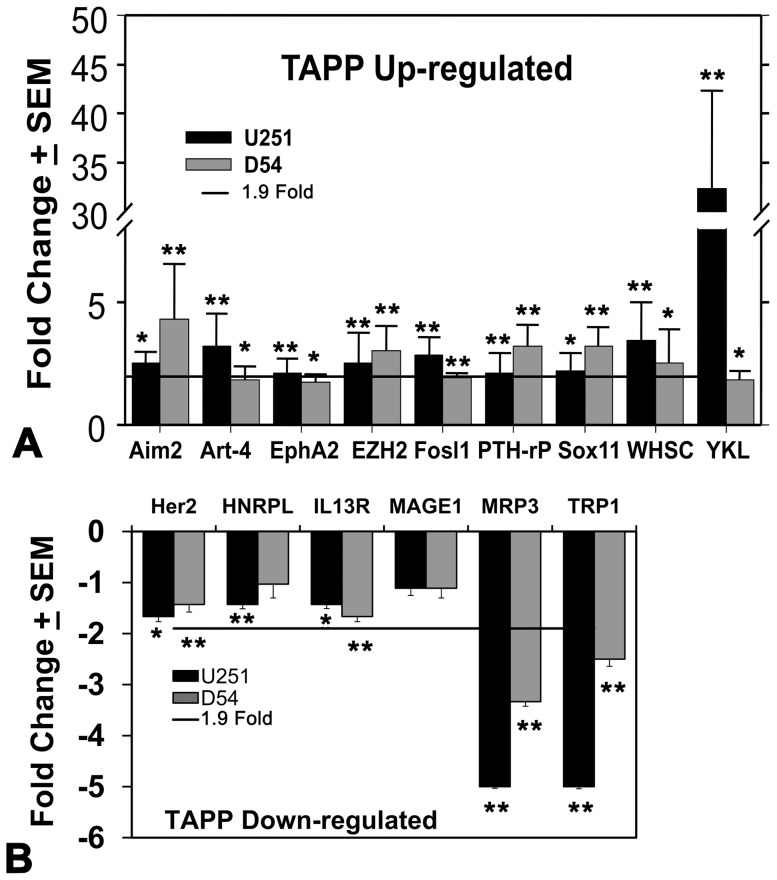
TAPP genes were differentially expressed in glioma cells grown under hypoxic conditions for one week. After one week of culture the hypoxic and normoxic grown cells were harvested, lysed and prepared for qRT-PCR analysis using the glioma tumor antigen panel. The cells grown under hypoxic conditions (1% O_2_) were directly compared to those simultaneously matched cells grown under normoxic conditions for 1 week. Samples were analyzed 3–6 times depending upon the closeness of the results. Data is represented as fold changes ± standard error of the means. Panel A shows TAPP genes that were up-regulated in both cell lines; the horizontal line represents the cut-off value (1.9-fold increase) that we believe represents a biologically significant value. Panel B shows the genes down-regulated in both glioma cells. The horizontal line in this panel represents a ≥−1.9- fold reduction value which we believe represents a threshold of biological significance. The data for the other TAPPs, which did not show any significant change, and is not presented for the sake of brevity. The single asterisk signifies P<0.05, while double asterisk shows P<0.01 value between the data generated from the normoxic and hypoxic cells.

Based on prior studies by others, we examined 25 possible glioma tumor-associated antigens. The significance of these antigens are 1) these glioma-associated antigens are documented to be expressed or over-expressed in human gliomas, 2) these antigens have induced human immune responses against any type of human cancer, 3) detection of these antigens' mRNA was amendable to our qRT-PCR technology [33], [34]. Figure 1 displays the TAPP mRNA profiles of the U251 and D54 glioma cells grown for 1 week under either hypoxic or normoxic conditions. We considered elevated levels of mRNA in the hypoxic glioma cells to occur when there was ≥1.9-fold expression over that displayed by the pair-matched normoxic controls. Figure 1A shows the profiles of TAPPs (Aim2, Art-4, EphA2, EZH2, Fosl1, PTH-rP, Sox11, Whsc2 and YKL-40) that were all elevated in the both glioma cell lines. All of these RNA levels were statistically significantly different from their normoxic control values. These values were deemed significant and are based on traditional microarray analysis done by others. Using these criteria, Aim2, Art-4, EphA2, EZH2, Fosl1, PTH-rP, Sox11, Whsc2 and YKL-40 were deemed to be biologically important. Most surprisingly, the U251 cells gave a very high response under hypoxia for YKL-40, while the D54 cellular response bordered only at the 1.9-fold level.

We used three other glioma cell lines, LNZ308, U87 and T98G, to show the universal nature of this increased qRT-PCR based response induced by hypoxia. The responses of the three most inducible antigens, EZH2, Whsc2 and YKL-40, were reproduced in these different glioma cell lines under hypoxia (Table 1).

**Table 1:** Three other human glioma cells make increased mRNA of EZH2, Whsc2 and YKL-40 under hypoxic conditions.

Antigen	Cell line	Fold increase
**EZH2**	LNZ308	3.0±0.6
	T98G	1.5±0.1
	U87	2.8±0.2
**Whsc2**	LNZ308	2.5±0.1
	T98G	1.6±0.4
	U87	1.8±0.4
**YKL-40**	LNZ308	3.8±0.5
	T98G	8.1±0.7
	U87	5.3±0.6

The human glioma cells (LNZ308, T98G and U87) were cultured at 1 million cells in 15 mls of media within T75 cm^2^ flasks either under hypoxic or normoxic conditions for 7 days. Afterwards, the mRNA was isolated and qRT-PCR was accomplished using the EZH2, Whsc2 and YKL-40 primers. Data is displayed as increased fold-differences of the hypoxic cells relative to the normoxic cells. The data represents the composite of three separate experiments and is presented as mean and the standard error of the means.

Figure 1B shows the genes that were down-regulated by hypoxia in both cell lines. The TAPPs include Her2, HNRPL, IL-13Rα2, MageA1/3/4/6, MRP3 and Trp1 were all somewhat down-regulated under hypoxic conditions. As we did for the over-expression, we also set cut-off values which could be considered biologically significant (≥−1.9 fold). MageA1/3/4/6 was slightly reduced by the hypoxic conditions (−1.11 fold reduction), but we believe this fails to be a medically significant reduction. Using this constraint, only MRP3 and Trp-1 were deemed biologically down-regulated and had (≥−2.3 fold reductions).

### Levels of EZH2, Whsc2 and YKL-40 protein are increased in hypoxic cells

**Figure 2:**
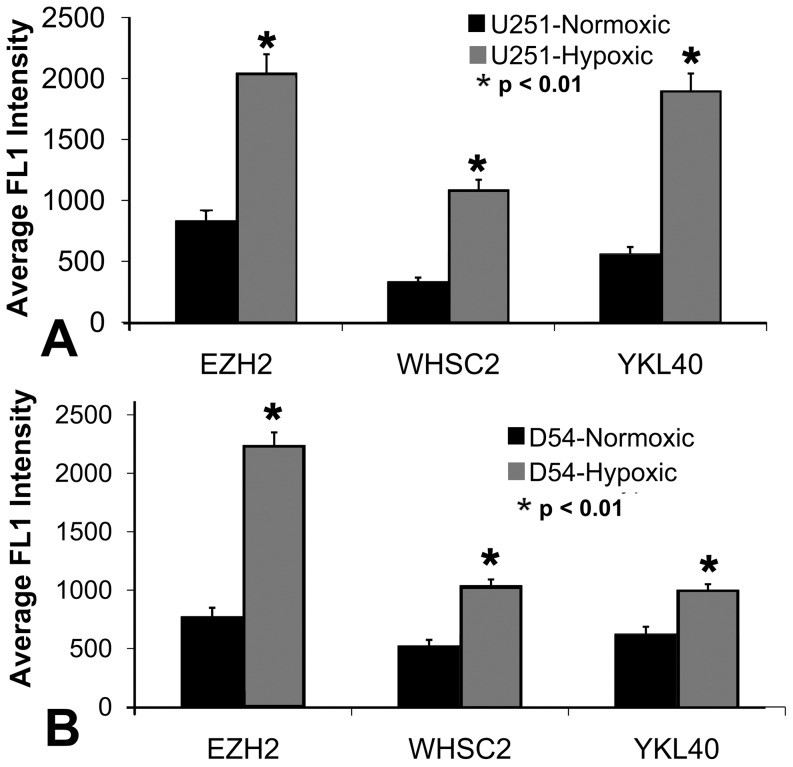
Glioma cells grown under hypoxic conditions express more EZH2, Whsc2 and YKL-40 at the protein level. U251 cells grown under either hypoxic or normoxic conditions for 1 week, were harvested, fixed and stained for the intracellular presence of EZH2, Whsc2 and YKL-40. Panel A displays the mean peak channel intensity of 10,000 U251 cells analyzed for intracellular EZH2, Whsc2 and YKL-40, while Panel B shows the data using the D54 cells. The asterisk indicates P<0.01 value.

We verified that the three most highly hypoxic-induced TAPP mRNA produced more protein expression of EZH2, Whsc2 and YKL-40 by doing either intracellular flow cytometry or immunofluorescent staining of hypoxic cells grown on coverslips. These three TAPPs were further investigated, since 1) commercial antibodies towards these antigens were available, 2) these tumor antigenic precursor protein mRNAs were highly expressed under hypoxic conditions, 3) these tumor antigens are still considered relatively new to glioma immunology. Figure 2A shows this increased protein expression of EZH2, Whsc2 and YKL-40 in the hypoxic U251 and D54 cells that was above the levels expressed by their corresponding normoxic cells. Using the Kolmogorov-Smirnov statistics test, the hypoxic response expression was significantly elevated (P<0.01) above that displayed by the normoxic control cells in both experiments. These experiments showing increased TAPP expression following hypoxia treatment were performed at least twice to show the reproducibility of this response.

To confirm the intracellular flow cytometry data, we used a quantitative electrophoretic analysis technique using the BioRad Experion to measure the amounts of these proteins contained in these hypoxic glioma cells. Table 2 summarizes the amount of the TAPP protein expressed in both normoxic and hypoxic cells. This data shows that these potential antigens were translated into protein and they were elevated in the hypoxic U251 and D54 cells. We also used two other glioma cells (T98G and LNZ308) to show identical results.

**Table 2:** Increased production of TAPP induced by hypoxia as determined by a quantitative analysis using the Bio-Rad Experion.

		Protein (ng/μl)		
Antigen	Cell Line	Normoxic	Hypoxic	Fold- Change
**EZH2**	U251	13.5	83.0	6.1
	T98G	9.8	22.5	2.3
	LNZ308	6.6	16.2	2.5
	D54	0.5	3.7	7.4
**Whsc2**	U251	93.4	170.5	1.8
	T98G	26.0	81.6	3.1
	LNZ308	35.5	55.3	1.6
	D54	22.9	65.3	2.9
**YKL-40**	U251	27.1	320.9	11.8
	T98G	23.4	80.6	3.4
	LNZ308	8.2	86.7	10.6
	D54	17.6	20.6	1.2

Human glioma cells (U251, T98G, LNZ308 and D54) were cultured for 1 week either under hypoxic or normoxic conditions. The cells were harvested, lysed and the protein concentration was determined by Lowry method. One µg/µl of total protein were run on the Pro260 protein chips and then quantitated. Recombinant EZH2, Whsc2 and YKL-40 were purchased from US Biologicals and used as the standard to calculate true protein concentrations.

**Figure 3:**
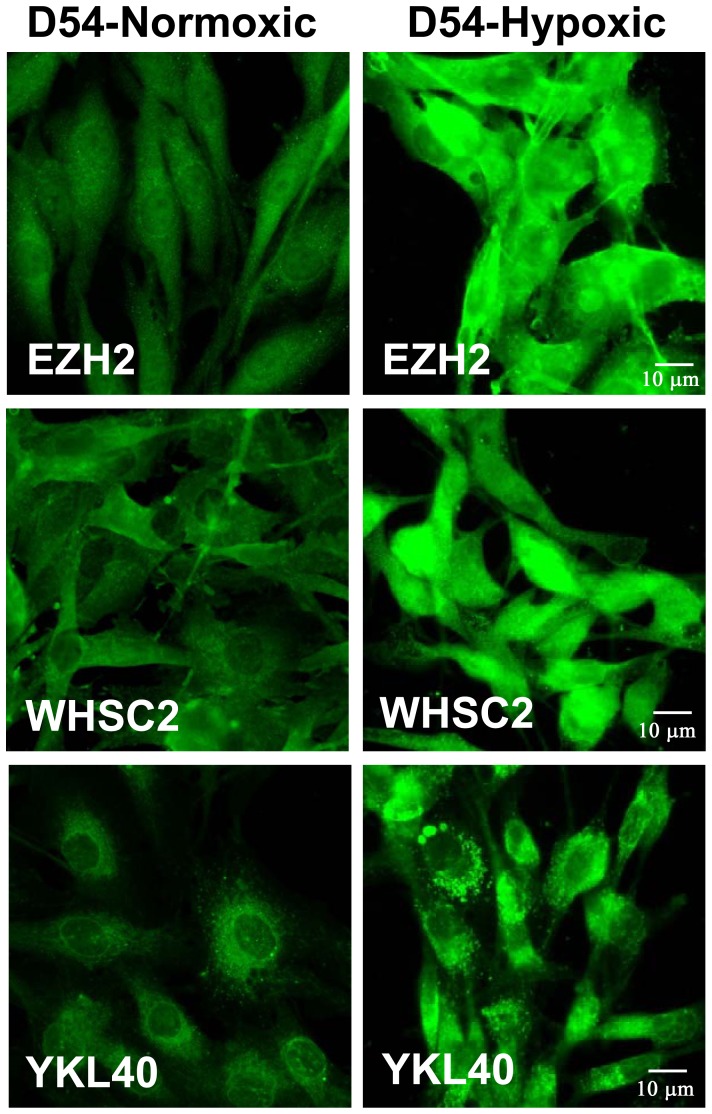
Immunofluorescent microscopy shows the up-regulated TAPP protein expression in the hypoxic D54 gliomas. D54 glioma cells were grown on a coverslips for 7 days either under normoxic or hypoxic conditions; the cells were fixed, permeabilized and stained with the primary antibodies (EZH2, Whsc2, and YKL-40). The cells were then visualized at 40×. The left column shows representative micrographs of normoxic cells, while the right column shows hypoxic cells.

We used immunofluorescent microscopic analysis to confirm that these induced proteins were present in the hypoxic D54 cells. Figure 3 displays increased expression in the hypoxic cells of all three up-regulated proteins. The top panels show baseline staining of the normoxic cells, while the bottom panels are representative of the hypoxic D54 cells. For all 3 TAPP staining profiles, there was increased staining intensity displayed by the hypoxic cells.

### Hypoxia-induced TAPP up- or down-regulation are reversible

**Figure 4:**
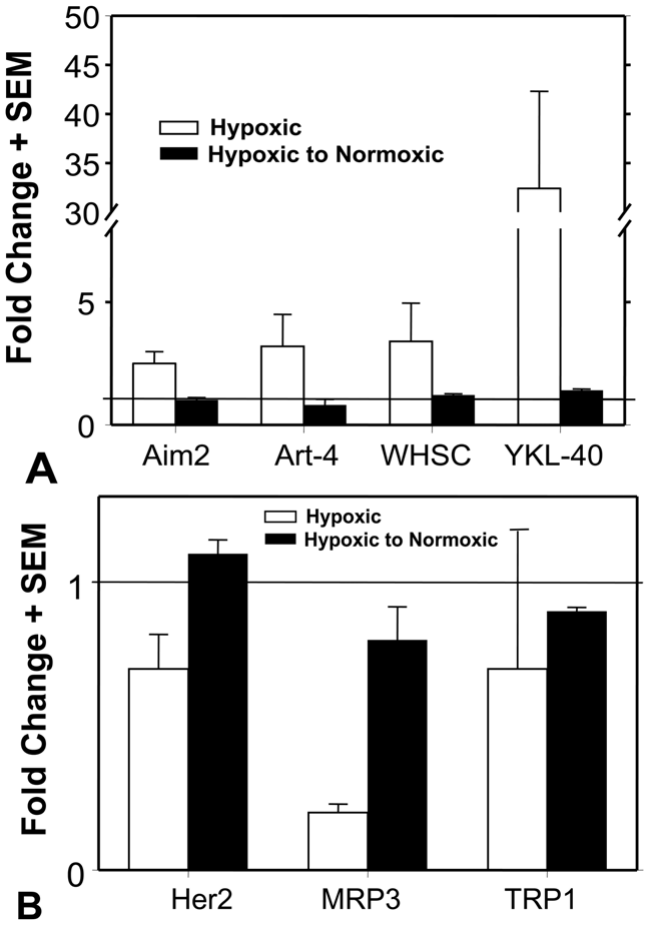
TAPP profiles grown under hypoxic condition are reversible after growing the cells for 1 week under normoxic conditions. U251 cells were grown under hypoxic conditions for 1 week were analyzed for qRT-PCR (designated as Hypoxic). An aliquot of these cells was continued to grow under hypoxic conditions for another 6 days (13 days total under hypoxic conditions). These cells were returned to normoxic conditions for one more week (designated as Hypoxic to Normoxic). These cells were compared to those U251 cells grown under normoxic conditions for 2 weeks. Panel A shows the genes that were up-regulated by hypoxia, while Panel B shows the genes that were down-regulated. The horizontal lines represent fold-change value of 1 which represents a baseline value of normoxic values.

U251 cells were grown under hypoxic conditions; they were passed at a1∶3 ratios after 1 week. Samples were analyzed for the real-time PCR values (labeled as Hypoxic in Figure 4) and confirmed the previous experiments in Figure 1A and 1B. After thirteen total days in a hypoxic environment, an aliquot of these hypoxic cells were re-cultured for 7-days back at normoxic conditions. Afterwards, these cells were tested for their mRNA profiles for those genes that were either up-regulated (labeled as Hypoxic to Normoxic). Figure 4A shows the data with the up-regulated genes: Aim2, Art4, Whsc2 and YKL40; Figure 4B shows the data with Her2, MRP3 and Trp1. The hypoxic cells that were re-cultured under normoxic conditions reverted back to their original TAPP mRNA values displayed when grown in normoxia.

### Hypoxia Inducible Factor-1α (HIF-1α) expression occurs at 1% O_2_ and also shows increased EZH2 and Whsc2 protein expression in hypoxic U251 cells

**Figure 5:**
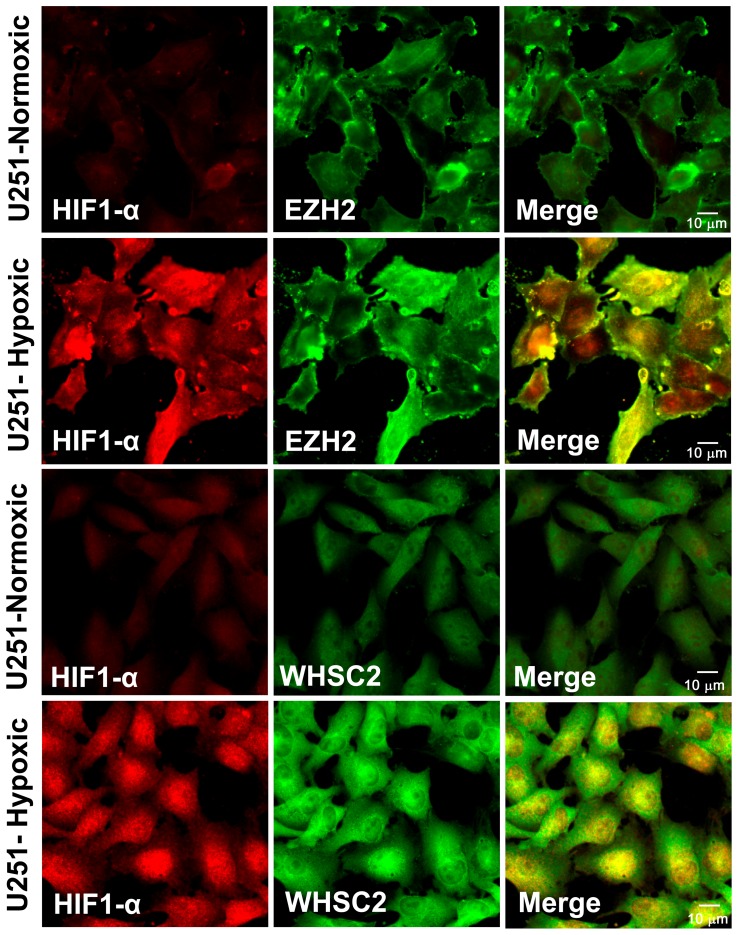
Hypoxia induces HIF-1α expression along with EZH2 and Whsc2 in hypoxic U251 cells as detected by confocal microscopy. U251 cells were grown on a coverslips for 7 days either under normoxic or hypoxic conditions, the cells were fixed, permeabilized and stained with the primary antibodies (HIF-1α, EZH and Whsc2). The cells were then visualized at 40× magnification. The Top Panels compare the normoxic and hypoxic pairings of HIF-1α and EZH2, while the Bottom Panels compare the HIF-1α and Whsc2 pairings.

We used the traditional transcription factor, hypoxia inducible factor-1α, to validate that hypoxia was being induced under our experimental conditions. U251 cells were cultured on glass coverslips and grown for 1 week. Afterwards the cells were fixed, permeabilized, and two-color staining was done with either anti-HIF-1α antibody staining (as detected by red staining) or anti-EZH2 or anti-Whsc2 antibodies (as detected by green staining). The two different fluorescent dyes in the stained sample were simultaneously visualized. The yellow color is the result of the both red emitting and green emitting probes being present with a close physical distance to each other. Figure 5 shows the results of these experiments. Under normoxic conditions, the U251 displayed minimal HIF-1α expression (red staining). Concurrently, the EZH2 and Whsc2 staining (green fluorescence) coming from these respective antibodies were positive, reflecting the baseline TAPP expression. Hypoxic U251 cells showed increased expression of HIF-1α, EZH2 and Whsc2 proteins when compared to the normoxic cells.

### Whsc2 and YKL-40 proteins are present in hypoxic regions of human glioma

**Figure 6:**
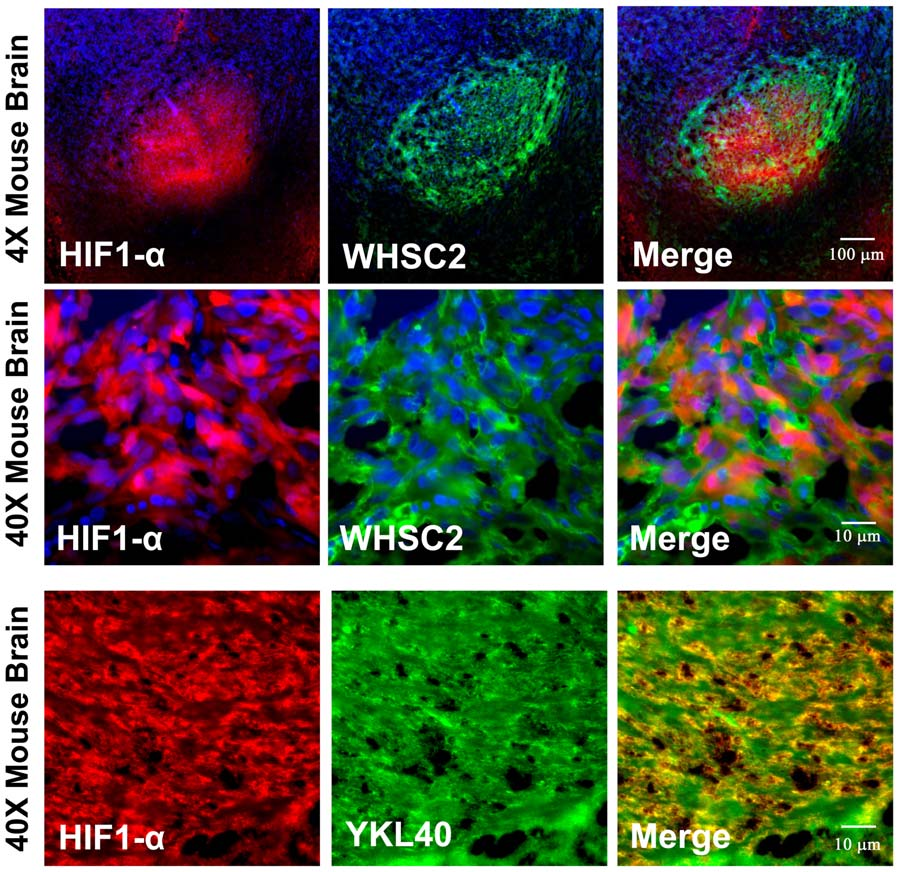
Two-color fluorescent microscopy shows co-localization of Whsc2 and YKL-40 with HIF-1α in U251 CD133+ “stem-like” cell induced tumors. Human U251 neurosphere “stem cells” were implanted into the brain of a nude mouse. When the glioma was showing an effect on the mouse, the mouse was euthanized and the brain was removed. Serial sections were cut and then stained with either anti-HIF-1α (red), Whsc2 (green) or YKL-40 (green). The nuclei are stained with DAPI (blue). The Top Panel Row shows a 4× magnification with HIF-1α and Whsc2, while the Middle Row Panels are taken with a 40× magnification. The Bottom Row Panels are the 40× magnification of HIF-1α and YKL-40 stained cells.

To test whether more clinically relevant GBM cells could reproduce this hypoxic effect, human GBM “stem-like” cells isolated from CD133+ U251 cells grown in neurosphere cultures for 5 months *in vitro* were used. These cells were surgically implanted into the brains of nude mice. After the mice began to show signs of disease, the mice were euthanized and the brains were removed. These brains were sectioned using a cryostat and were immunostained with anti-HIF-1α antibody (green) to identify the regions of hypoxia. HIF-1α expression maximally occurs *in vivo* at 1.5–2% O_2_. [37]. Human glioma tissue was co-stained with either anti-Whsc2 or anti-YKL-40 antibodies (detected by red staining) to show co-localization within the same cells. Immunohistochemistry revealed a more invasive histology that is more characteristic of human clinical GBM. Figure S1 shows the invasiveness of this glioma more clearly in three peripheral locations (Panel A). Panel B shows a magnified region of one of the regions where infiltration is prominently observed. Figure 6 shows two-color immuno-fluorescent microscopy of a human glioma growing *in situ*. In general, where there were regions of HIF-1α staining, we also saw co-localization of either Whsc2 or YKL-40 staining (yellow staining). The intensity of staining with Whsc2 or YKL-40 appeared stronger than that of HIF-1α expression. The yellow color indicates that co-localization of these proteins is occurring, denoting that the induced proteins were physically located very close to each other.

### MHC class I expression remains on U251 cells cultured under hypoxic conditions

**Figure 7:**
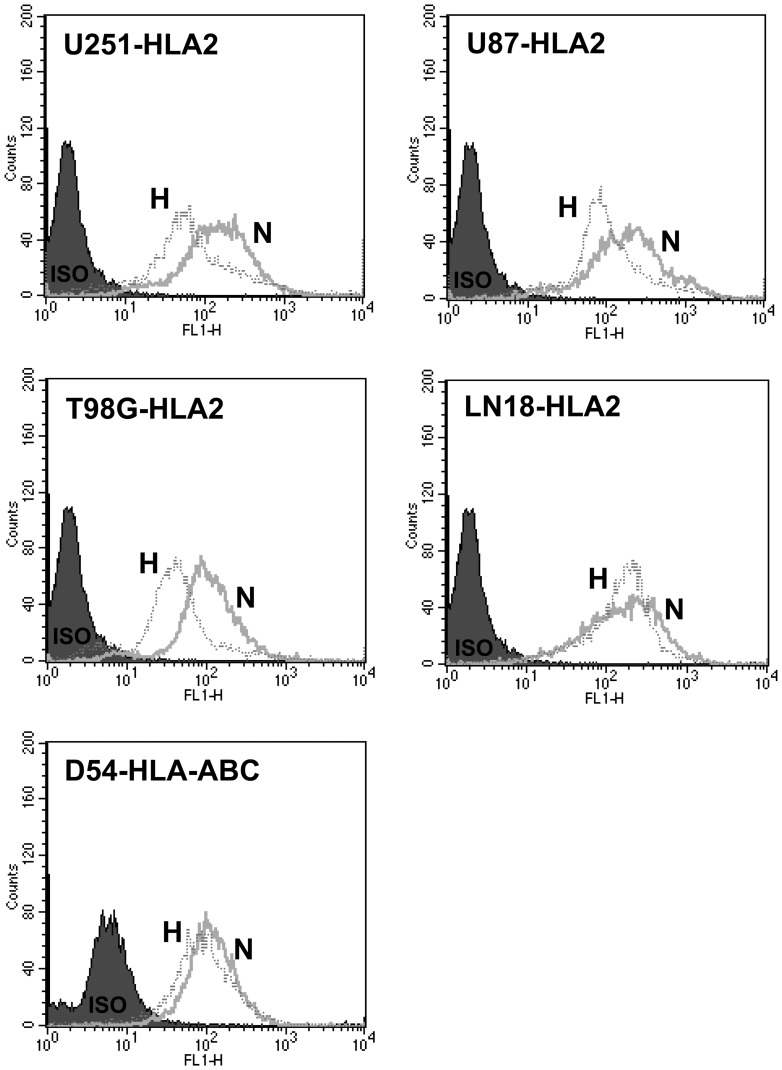
Comparison of HLA-Class I profiles of human glioma cells grown under hypoxic or normoxic conditions. The U251, U87, T98G, LN18 or D54 glioma cells were either grown under normoxic or hypoxic conditions for 1 week. The cells were stained for either cell-surface HLA-A2 (U251, U87, T98G and LN18) or HLA-ABC framework (D54) specific antibodies. Ten thousand cells were analyzed by flow cytometry. The profile or the hypoxic cells (labeled H, indicated by heavy line) is compared to the shaded normoxic cells (labeled N, indicated by thin line). The isotypic controls of the normoxic and hypoxic cells are superimposable and are shown as a single black shaded profile.

Hypoxic cells expressed similar amounts of major histocompatibility antigens (HLA class I molecules) as the normoxic cells. Figure 7 shows representative HLA-A0201 or HLA-ABC framework expression of five human glioma cells (U251, U87, T98G, LN18 and D54) that was done three to four times. The expression of these MHC molecules on the tumor cells should sufficiently allow the tumor antigenic peptides to be recognized by the appropriate T cells.

### CTLs kill hypoxic U251 cells more effectively than normoxic cells

Our data here indicated that the various glioma cell lines made increased amounts of the TAPPs. The presence of the whole precursor protein does not guarantee that these antigens are properly processed into the antigenic peptides that CTLs can functionally respond to on the MHC class I molecules. We demonstrated that functional glioma antigens on hypoxic cells are better recognized by CTL effector cells by doing cell-mediated cytolysis experiments. Since U251 cells are HLA-0201 [33], we found a peptide that was derived from the Whsc2 TAPP that could be recognized by CTLs via a HLA-0201 dependent restriction. The peptide, Whsc2 _103–111_ (ASLDSDPWV) [38], [39] was incubated with the immature dendritic cells and then the matured DC were used to stimulate *in vitro* human CD8 CTL responses. These CTLs were then used to determine whether this difference in TAPP expression has biological significance.

**Figure 8:**
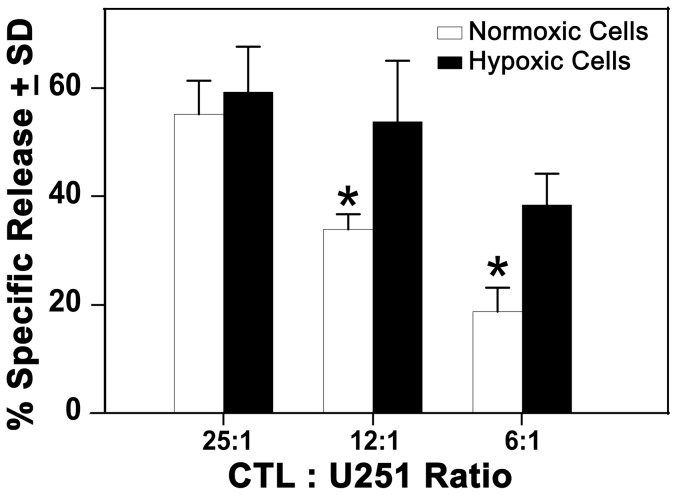
Hypoxic U251 cells are killed better by Whsc2 specific human CTLs. HLA-A0201 restricted CTLs were generated *in vitro* against the Whsc2 _103–111_ peptide. The U251 target cells were either grown under normoxic or hypoxic conditions for 1 week. The target cells were radio-labeled with Cr^51^. The lymphocytes were incubated with the target cells for 6 hrs at 37C in quadruplicate cultures. The data is presented as % Specific Release ± Standard Deviation (SD). Asterisks indicate P<0.05 as determined by a Student's t test to show the statistical differences between the cytotoxic data at either the respective 12∶1 or 6∶1 effector:target ratios.

Figure 8 shows that the anti-Whsc2-specific CTL killed the hypoxic or normoxic cells, under normoxic conditions in a dose-dependent relationship. At the 25∶1 effector: target ratio, the CTLs equally killed the normoxic or hypoxic U251 cells. However, the CTLs killed the hypoxic U251 cells better when the lower 12∶1 and 6∶1 effector: target ratios were used. We interpret this response to mean that more HLA-A2 molecules were loaded with the additional Whsc2 peptides, even though there were fewer HLA molecules on the hypoxic cells. So a higher percentage of HLA-A2 molecules were recognized by the anti-Whsc2 specific CTLs at the lower E:T.

## Discussion

Human glial cell tumors express many tumor associated antigens that can stimulate human T cell immune responses either *in vitro* or *in vivo* [16] [Bibr pone.0042661-Kruse2] [Bibr pone.0042661-Yu1] [Bibr pone.0042661-Yu2] [Bibr pone.0042661-Liau1] [Bibr pone.0042661-Kim1] [Bibr pone.0042661-Yamanaka1] [Bibr pone.0042661-Hickey1] [Bibr pone.0042661-Ge1] [Bibr pone.0042661-Grauer1] [Bibr pone.0042661-Toda1] [Bibr pone.0042661-Heimberger1] [Bibr pone.0042661-Izumoto1] [Bibr pone.0042661-Parney1]–[30] as summarized in Table S1. DC vaccination with the antigens/lysates from the “mesenchymal subtype” of GBM produced a better survival than those other types of GBM treated with their respective lysate-pulsed DC; i.e., classical or pro-neural types [40]. Mesenchymal-type GBMs have a very poor prognosis when compared to the pro-neural GBM [41], [42]. Thus, finding tumor antigens which the lymphocytes of GBM patients can respond to is vitally important.

We examined 25 glioma-associated tumor antigenic precursor proteins (TAPP) in 2 well-known human GBM cell lines (U251 and D54) as measured by using quantitative reverse transcriptase, real-time polymerase chain reactions (qRT-PCR). These 25 TAPP were chosen based on extensive literature searching for the ability of these gliomas derived molecules to elicit human T cell immune responses (summarized in Table S1). Many of the TAPP were not differentially expressed in the hypoxic cells but nine TAPPs from the glioma cells (Aim2, Art-4, EphA2, EZH2, Fosl1, PTH-rP, Sox11, Whsc2 and YKL-40) consistently exhibited increased mRNA presence after culturing the cells in response to hypoxia (Figure 1A). Two other antigens (MRP3 and Trp-1) showed decreased mRNA expression in response to 1% hypoxia (Figure 1B). These results demonstrate the variable effects of hypoxia on the expression of TAPPs which may also be reflected in the tumor microenvironment.

Three of the most hypoxia-inducible tumor antigen precursor proteins (EZH2, Whsc2 and YKL-40) were confirmed to be up-regulated at the protein level by hypoxia (Figures 2 and 3). This effect was confirmed in other glioma cell lines U87, T98G, and LNZ308 (Tables 1 and 2). The altered TAPP expression in cells grown under hypoxic conditions returned to normal baseline values when the cells were returned to normoxic conditions. This finding illustrates that these hypoxic-specific responses are reversible (Figure 4). Thus, tumor cells grown under hypoxic conditions are not being selected for stable variants that possessed a different TAPP phenotype. The data shown here indicate that the tumor antigen profiles do have an oxygen dependent characteristic. Under normoxic conditions, the U251 cells displayed minimal HIF-1α expression with slightly positive staining for the EZH2 and Whsc2. When compared to the hypoxic U251 cells, the expression of HIF-1α, EZH2 and Whsc2 was increased and co-localized to the same cells, indicating this induction occurred in the same cells and not just in random cells. We also showed that U251 “CD133+ stem-like” cells when implanted into a nude mouse brain successfully reproduced the hypoxic profile as indicated by co-localized HIF-1α staining (Figure 6) with increased Whsc2 and YKL-40 staining. The *in vivo* evidence does closely reproduce the data we saw in the *in vitro* hypoxic conditions using U251 neurosphere-derived cells.

Growing human glioma cells under hypoxia had only minimal effects of MHC class I expression (Figure 7). Human CTLs specific for the Whsc2_103–111_ peptide demonstrated enhanced killing on the hypoxic U251 (Figure 8), indicating that the increased hypoxic expression can have biological significance. The increased quantities of these peptides theoretically can be loaded onto the HLA-A2 molecules and allow better T cell recognition. However, we can't fully exclude other unknown intrinsic biochemical effects occurring during hypoxia that explains the increased cytotoxicity displayed by the hypoxic cells responding to the Whsc2 specific CTLs. These results suggest that the source of antigenic material may have a profound effect upon generating effective immune responses, not only in generating CTLs but also in targeting certain cancers' antigens within the tumor. Hypoxic tumor cells do express more of certain TAPPs and can at least in the case of one antigen, Whsc2_103–111_ be attacked, once the primed CTLs become activated CTLs *in vivo* by the DC-vaccination.

Wolf-Hirschhorn syndrome candidate 2 (Whsc2) is a member of the NELF (negative elongation factor) protein complex that participates in the regulation of RNA polymerase II transcription elongation [43], [44]. This gene is commonly found to be expressed in fetal tissue and gradually loses its expression in adults. This protein is commonly found to be truncated in various cancers including gliomas [38], [39]. EZH2 (Enhancer of Zeste 2) belongs to the polycomb transcription factor family of proteins and is associated with various cancer stem cells, including human gliomas [45], [46]. The exact roles of these two proteins (Whsc2 and EZH2) in gliomagenesis are still unknown. It is possible that by targeting these TAPP, activated CTLs might also be attacking the GBM “stem-like cells”. YKL-40 is also known to be produced in larger amounts in advanced gliomas; these patients have a worse prognosis [47], [48]. It has been reported that when gliomas possess a high amount of hypoxic regions, those patients have a poorer survival rate when compared to lower amounts of hypoxia [9], [12]. It not surprising that YKL-40 is involved in this pathological process. YKL-40 has been reported to be an angiogenic factor and could help play a role in brain tumor vasculogenesis [49]. The expression of YKL-40 was much more noticeable in the U251 cell than in the D54 cells. YKL-40 (also known as chitinase-3-like protein-1, CHI3L1) is heavily expressed at the protein level in the glioma proteome [50]. Epitopes from this molecule are associated with the HLA-A2+ gliomas and can induce human CTL responses [30]. This molecule could reflect the unique energetic characteristics (glycolytic and oxidative phosphorylation) of the U251 cells [35], [36], or YKL-40 is a marker of the mesenchymal subtype of GBM [41], so the U251 cells could be a representative cell line of this GBM subtype.

In terms of human immune responses towards these three glioma-associated antigens, in 2008, Zhang, et al., [34] showed that these 3 TAPP mRNA were highly expressed by a variety of human gliomas. YKL-40 was equally seen in human GBM derived from both adults and pediatric patients. EZH2 and Whsc2 were heavily expressed in adult GBM, while weakly expressed in pediatric GBM. CTLs were generated against YKL-40 antigenic peptides and those CTLs could kill GBM cell lines [51]. Clinically, this antigen has been recently added into therapeutic protocols against human gliomas [52]. Dendritic cells pulsed with this peptide in a cocktail elicited responses from patients' lymphocytes responding to antigen-pulsed DC by positive ELISPOTs. Whsc2 mRNA was initially discovered to be made by gliomas and CTLs could be developed that responded towards glioma cells [39], [53]. By proteomic studies and confirmed by western blotting, Whsc2 was found to be heavily expressed in gliomas, but not in normal tissue [54]. EZH2 is up-regulated in gliomas and GBM stem-like cells [55]. Yajima, et al. [39] showed EZH2 expression in GBM cells could be the targets of CTLs. Thus, these 3 tumor antigens may represent useful targets in an activated immune response for human glioma therapy.

In summary, we show that nine possible glioma tumor antigens out of 25 antigens tested were up-regulated and two tumor antigens were down-regulated by hypoxia as detected by qRT-PCR. We confirmed that the most highly hypoxia-induced TAPP did have increased protein expression *in vitro*. These same antigens were also found *in situ* using isolated GBM-derived neurosphere cells that formed intracranial tumors in nude mice. One week cultured hypoxic U251 cells were better targets for Whsc2-specific CTLs than were normoxic grown U251 cells. Thus, hypoxic cells may provide a somewhat better source of tumor antigens for stimulating human immune responses for certain tumor-associated antigens via dendritic cells. Hence hypoxic cells might actually be preferable for stimulating immune responses for many glioma-associated antigens.

## Materials and Methods

### Cell lines and cell culture

U251 was obtained from Dr. Dennis Deen (University of California, San Francisco, CA). Dr. Carol Kruse of the Sidney Kimmel Cancer Center (San Diego, Ca) supplied us with the D54 as previously described [33]. The LN-18 cells were purchased from the American Type Culture Collection (Manasas, VA). The U87, T98G and LNZ308 were obtained from Dr. Thomas Chen (Univ. of Southern California, Los Angeles, CA). The glioma cells were grown in DMEM media (Sigma Chemical Comp., St. Louis, MO) supplemented with 5% fetal bovine serum (FBS) (Gemini BioProducts, Calabassas, CA). Peripheral blood from healthy normal HLA-A2+ donors was obtained after informed consent was signed at Hoag Hospital. These human blood collections were previously approved by the Institutional Review Board from Hoag Hospital Memorial Presbyterian Hospital Comprehensive Cancer Center, when Dr. Andrew Cornforth was previously employed there.

### Hypoxic growth conditions

Cells were initiated under identical conditions, one aliquot of the cell culture was placed within paired standard vented T-25 cm^2^ or T-75 cm^2^ flasks. One flask was cultured using the standard tissue culture incubator 37°C, 5% CO_2_ (normoxic conditions), while the identical matched flask was placed within a Coy Laboratory Products (Grass Lake, MI) hypoxic chamber possessing 5% CO_2_ and 1% O_2_, with the balance being N_2_. These cells were incubated at 37°C in a Pro-blot portable incubator (Labnet, Woodbridge, NJ) contained within the hypoxic workstation.

### Real-Time Polymerase Chain Reaction Analysis

Total RNA was isolated from the cells using RNeasy Plus mini kit from Qiagen (Valencia, Ca). cDNA was synthesized using iScriptTM cDNA synthesis Kit (Bio-Rad Laboratories, Hercules, CA) containing 1 µg total RNA/sample. Real-time PCR reactions were performed on an iCycler iQ detection system (Bio-Rad Laboratories, Hercules, CA) in conjunction with the SYBR Green kit (Bio-Rad Laboratories, Hercules, CA). The thermal profile was 95°C for 15 min, followed by 40 cycles of 95°C for 15 s and 58°C for 30 s, finally holding at 4°C. The primers for all 25 glioma-associated tumor antigens were synthesized by Operon Biotech (Huntsville, AL). The sequences of these primers are found in Table S2.

Samples were run in quadruplicate, and a reaction without cDNA was used to establish baseline fluorescence levels with 18 S RNA. The fluorescent signal was plotted versus cycle number, and the threshold cycle (Ct) was determined when the cycle number, where an increase above background fluorescence could be reliably detected. Each PCR run also included non-template controls containing all reagents except cDNA. After cycling, a melting curve was produced by slow denaturation of the PCR end products to validate the specificity of amplification. The relative quantification of expression of the gene was determined by 2^−ΔΔCT^ as described by Pfaffl [33], [34], [56].

Data showing fold increases as a result of hypoxia is presented as fold increases ± Standard Error of the Means (SEM). For data that shows a reduction of mRNA, we present this same data as the negative inverse of the raw fold induction (e.g. 0.2-fold induction is presented as a −5 fold induction) to achieve a negative fold increase of expression. For both sets of data, a value of 1.9 fold induction or reduction is considerate significant.

### Antibodies

The glioma cells were stained for either the specific HLA-A2 allele (cat # 551230) or for the HLA-ABC framework (cat # 557347) using the mouse monoclonal antibodies purchased from Pharmingen BD Biosciences (San Diego, CA). Mouse anti-human Whsc2 and anti-human EZH2 antibodies were purchased from Novus Biologicals (Littleton, CO). Mouse anti- human YKL-40 and rabbit anti-human YKL-40 antibodies were purchased from Quidel Corp. (San Diego, CA). Anti-human HIF-1α antibody was purchased from Santa Cruz Biotechnology (Santa Cruz, CA).

### Intracellular Flow Cytometry

Glioma cells (10×10^6^) were prepared with the reagents and protocols of Santa Cruz Biotechnology (Santa Cruz, CA). The fixed cells were washed twice in ice-cold PBS. The cells were permeabilized for 15 minutes on ice. The cells were washed twice. The resuspended cells were then divided into 10^6^ cell aliquots and then incubated with the primary antibody for 1 hr. The cells were washed twice and the secondary antibody-conjugated with fluorescein isothiocyanate (FITC) (Vector Labs, Burlingame, CA) was incubated on ice for another hour. After washing the cells twice, ten thousand cells were analyzed with a Bectin Dickinson FACS Calibur flow cytometer (Mountain View, CA). We used the Kolmogorov-Smirnov statistics test, to show significant differences in flow cytometry expressions (P<0.05).

### Quantitation of EZH2, Whsc2 and YKL-40 protein expression

Human glioma cells were cultured in T-75 cm^2^ flasks for 1 week either under hypoxic or normoxic conditions as described above. The cells were harvested, lysed in RIPA buffer and the protein concentration was determined by the Bradford method (Bio-Rad Laboratories, Hercules, CA). One microgram/microliter of total protein from each cell lysate was analyzed on the Bio-Rad Experion (Hercules, CA) Pro260 microfluidic protein chips, according to the manufacturer's directions. The Experion Pro260 protein ladder was used as the molecular weight standards. Full length recombinant EZH2, was purchased from US Biologicals (Boston, MA). To generate the calibration curve, five known concentrations (100 ng/µl, 50 ng/µl, 25 ng/µl, 12.5 ng/µl, and 6.25 ng/µl) of the protein EZH2 were loaded into different sample wells and electrophoresis was performed with glioma samples. After electrophoresis was completed, the standard curve was generated and Ezh2 protein quantity was calculated. Whsc2 and YKL-40 in glioma cell lysates were quantified using relative quantification. The Experion software provides an estimate of protein concentration by comparing the ratio of the corrected area of each protein to that of the internal standard, which is present at a single, known concentration in the sample. Data is presented as ng/ml.

### Peptides

The peptide: Whsc2_103–111_: ASLDSDPWV was synthesized by Gen Script Corporation (Piscataway, NJ). This was based upon a prior study by Itoh, et al., [38] and Yajima, et al., [39] who showed a HLA-A0201 restriction to this peptide and Whsc2 sequences were detectable in human GBM.

### Generation of Cytotoxic T lymphocytes

Peripheral blood mononuclear cells were collected from normal non-cancer HLA-A2+ donors after informed consent forms were signed at Hoag Hospital. The mononuclear cells were ficolled and then incubated in human GM-CSF (1,000 units/ml) and IL-4 (1,000 units/ml) in AimV (Gibco/Invitrogen, San Diego, CA) at 37°C. The recombinant cytokines were obtained from CellGenix USA (Antioch, Illinois). In unpublished studies, by reverse transcriptase- real time polymerase chain reactions, U251 cells made Whsc2 mRNA and its protein was confirmed by intracellular flow cytometry using the Novus Corporation's antibodies (Littleton, CO). The Whsc2_103–111_ peptide, ASLDPWVL, was incubated with the 6 day old dendritic cells. The immature DCs were stimulated with recombinant human TNF-α, IL-6 and IL-1ß (10 ng/ml each). Mature DCs were harvested on day 8, resuspended in AIM-V medium at 10^6^ cells/ml with peptide (10 µg/ml), and incubated for 2 hr at 37°C. Populations of CD8+ T cells, autologous to the DC donors, were enriched from PBMC using magnetic microbeads (Miltenyi Biotech, Auburn, CA). CD8+ T cells (2×10^6^/well) were co-cultured with peptide-pulsed DCs (2×10^5^/well) in 2 ml of AIM-V medium supplemented with 5% human AB serum (Invitrogen, San Diego, CA), 10 units/ml rhIL-2 (R&D Systems, Minneapolis, MN), and 10 units/ml rhIL-7 (Cell Sciences, Canton, MA) in each well of 24-well tissue culture plates. On day 15, to increase CTL frequency, the lymphocytes were restimulated with autologous DCs pulsed with peptide in AIM-V medium supplemented with 5% human AB serum (Life Technologies, San Diego, CA), rhIL-2, and rhIL-7 (10 units/ml each).

The CTLs were used to test their potential cytolytic activity against U251 cells that were grown either under normoxic conditions of hypoxic conditions (1 week of culture). The lymphocytes were plated in V-bottom 96-well plates and were serially diluted at 1∶2 ratios. The tumor cells (10^4^ cells/well) were then added. The plates were incubated under normoxia at 37°C for 6 hr. Percent specific release was calculated using the equation described below for cytotoxicity.

% Specific killing = CPM experimental- CPM spontaneous release.

CPM maximum release- CPM spontaneous release×100.

The tumor cell killing is presented as mean values ± standard deviations.

Cytotoxicity data from quadruplicate cultures at each effector cell: tumor cells ratio are presented as mean specific killing ± standard deviation. Values were considered significantly different if P<0.05 using the Student's t test.

### Immunohistology of human neurosphere-induced tumors in nude mice

Two U251 cell lines were isolated from CD133+ cells and were grown in culture in neural sphere culture conditions. While under surgical anesthesia (1×10^5^ cells in a volume of 5 µl) were implanted into the frontal lobes of nude mice obtained from NCrNu-M from Taconic farms (Hudson, NY). This protocol was approved under the University of California, Irvine's Institutional Animal Care and Use Committee given to Dr. Zhou. Once the mice showed signs of tumor growth, the mice were euthanized and the brains were removed. The tissues were prepared as frozen sections.

Fresh xenograft-baring mouse brains were removed, fixed in 4% paraformaldehyde at 4 C over night, transferred to 30% sucrose to dehydrate over night, and mounted in OCT on dry ice. The cryosections (7–8 µm) were mounted on cleaned glass slides for immunostaining. Similarly, paraformaldehyde fixed U251 and D54 cells grown on cover glass and tissue section slides were washed four times in 0.01 M phosphate buffer saline (PBS). To quench non-specific binding, sections were incubated in 10% v/v normal donkey serum (in PBS-triton 0.3% v/v (PBST)) for 1 hour at room temperature. Sections were then probed with 10 µg/ml rabbit HIF-1α (Santa Crux Biotech, Santa Cruz, CA), or the mouse antibody specific for human Whsc2 (15 µg/ml) (Novus Corporation, Littleton, CO), or the mouse antibody specific for human YKL40 (15 µg/ml) (Novus Corporation, Littleton, CO) overnight at 4°C. Tissue sections were washed three times in 0.01 M phosphate buffer saline (PBS) then were incubated for two hours at room temperature with appropriate secondary antibody donkey anti-mouse, rabbit, rat, or goat Alexa Fluor 488 (green), Texas Red, and with or without nucleus counter stain Hoechst 33342 (Invitrogen, San Diego, CA). Finally, the tissue sections were washed three times and mounted with ProLong Gold antifade reagent (Invitrogen, San Diego, CA). Samples were acquired with fluorescence microscope equipped with RT Spot camera and software from Diagnostic Instrument (Sterling Heights, MI), and co-localization images were captured and analyzed with the Nikon two lasers (HeNe and Argon) PCM 2000 Confocal Eclipse E800 Nikon Microscope System. Yellow color shown on images were the result of displaying the two channels green and red fluorescence probes of the same sample at the same time by using simultaneous multi-channel Confocal imaging technique. Thus, it can be inferred that these proteins reside within very close spatial locations to each other.

## Supporting Information

Table S1 **Glioma-associated tumor antigens and key immunological information.**
(DOCX)

Table S2 **qRT - PCR primers used for this study.**
(DOCX)

Figure S1 **Two-color fluorescent microscopy shows co-localization of Whsc2 and YKL-40 with HIF-1α in U251 CD133+ “stem-like” cell induced tumors.** Human U251 neurosphere “stem cells” were implanted into the brain of a nude mouse. When the glioma was showing an effect on the mouse, the mouse was euthanized and the brain was removed. Serial sections were cut and then stained with either anti-HIF-1α (red), Whsc2 (green) or YKL-40 (green). The nuclei are stained with DAPI (blue). Panel B shows a magnified region that was highlighted from Panel A.
(TIF)
