# Supplementary material for: Differential Glioma-Associated Tumor Antigen Expression Profiles of Human Glioma Cells Grown in Hypoxia
Source: PLoS One. 2012 Sep 5;7(9):e42661. doi: 10.1371/journal.pone.0042661 (PMC3434178; doi:10.1371/journal.pone.0042661)
Supplement: Table S1 — Glioma-associated tumor antigens and key immunological information. (DOCX) [file pone.0042661.s001.docx]

| **Table S1:  *Glioma-associated tumor antigens and key immunological facts*** |
| --- |

| **Symbols** | **Descriptions** | **Key Findings** |
| --- | --- | --- |
| ***AIM2*** | *Antigen Isolated from Immunoselected Melanoma 2* | Initially, this tumor antigen was found within melanoma cells. The transcript is an open reading frame and comes from 2 spliced variant forms. H. Mamoru; Y. Li, M. El-Gamil, G.A. Ohnmacht, S.A. Rosenberg, and P.F. Robbins. 2001. Melanoma-Reactive CD8 + T Cells Recognize a Novel Tumor Antigen Expressed in a Wide Variety of Tumor Types. J Immunother 24: 323-333. Since melanomas and glioma share a common progeny cell. This antigen was subsequently discovered to be expressed within glioma cells. HLA-A1 restricted and CTLs were able to recognize this antigen when it is expressed within human gliomas. G. Liu, J.S. Yu, G. Zeng, D, Yin, D. Xie, K.L. Black, and H. Ying. 2004. AIM-2: A novel tumor antigen is expressed and presented by human glioma cells. J. Immunotherapy: 2004, 27: 220-226. Surgically resected adult and pediatric tissue can express this antigen at the mRNA level. Zhang JG, E. Newcomb, D. Zagzag, C. Kruse, L. Driggers, N. Hoa, M.R. Jadus. 2008. Tumor antigenic profiles of adult and pediatric brain tumors. J. Neuro-Oncol. 88:65-76. |
| ***ART-1*** | *Antigen Recognized by T cells-1 or ADP-ribosylation factor 1* | This antigen was defined by T cell clones that this Japanese group discovered. These antigens were found to have cross-reactive with various tumors, including gliomas and functional CTLs assays showed activity. N. Nonaka, S. Tsuda,^,^ S. Shichijo, M. Ito, Y. Maeda, M. Harada, T. Kamura, M. Shigemori, and K. Itoh. 2002 Recognition of ADP-ribosylation factor 4-like by HLA-A2-restricted and tumor-reactive cytotoxic T lymphocytes from patients with brain tumors. Tissue Antigens. 60:319–327. Surgically resected adult and pediatric tissue can express this antigen at the mRNA level. Zhang JG, E. Newcomb, D. Zagzag, C. Kruse, L. Driggers, N. Hoa, M.R. Jadus. 2008. Tumor antigenic profiles of adult and pediatric brain tumors. J. Neuro-Oncol. 88:65-76. |
| ***ART-4*** | *Antigen Recognized by T cells-4 ) or*  *ADP-ribosylation factor 4* | This antigen was definded by T cell clones that this Japanese group discovered. These antigens were found to have cross-reactive with various tumors, including gliomas and functional CTLs assays showed activity. N. Nonaka, S. Tsuda,^,^ S. Shichijo, M. Ito, Y. Maeda, M. Harada, T. Kamura, M. Shigemori, and K. Itoh. 2002. The same group also showed similar data with different tumors. Y. Nonaka^,^N. Tsuda^,^ S. Shichijo, M. Ito^,^  Y. Maeda, M. Harada, T. Kamura, M. Shigemori, and K. Itoh. 2002. Recognition of ADP-ribosylation factor 4-like by HLA-A2-restricted and tumor-reactive cytotoxic T lymphocytes from patients with brain tumors. Tissue Antigens. 60:319–327.Surgically resected adult and pediatric tissue can express this antigen at the mRNA level. Zhang JG, E. Newcomb, D. Zagzag, C. Kruse, L. Driggers, N. Hoa, M.R. Jadus. 2008. Tumor antigenic profiles of adult and pediatric brain tumors. J. Neuro-Oncol. 88:65-76. [N. Yajima](http://clincancerres.aacrjournals.org/search?author1=Naoki+Yajima&sortspec=date&submit=Submit), [R. Yamanaka](http://clincancerres.aacrjournals.org/search?author1=Ryuya+Yamanaka&sortspec=date&submit=Submit), [T. Mine](http://clincancerres.aacrjournals.org/search?author1=Takashi+Mine&sortspec=date&submit=Submit), [N. Tsuchiya](http://clincancerres.aacrjournals.org/search?author1=Naoto+Tsuchiya&sortspec=date&submit=Submit), [J. Homma](http://clincancerres.aacrjournals.org/search?author1=Jumpei+Homma&sortspec=date&submit=Submit), [M. Sano](http://clincancerres.aacrjournals.org/search?author1=Masakazu+Sano&sortspec=date&submit=Submit), [T. Kuramoto](http://clincancerres.aacrjournals.org/search?author1=Terukazu+Kuramoto&sortspec=date&submit=Submit), [Y. Obata](http://clincancerres.aacrjournals.org/search?author1=Yayoi+Obata&sortspec=date&submit=Submit), [N. Komatsu](http://clincancerres.aacrjournals.org/search?author1=Nobukazu+Komatsu&sortspec=date&submit=Submit), [Y. Arima](http://clincancerres.aacrjournals.org/search?author1=Yoshimi+Arima&sortspec=date&submit=Submit), [A. Yamada](http://clincancerres.aacrjournals.org/search?author1=Akira+Yamada&sortspec=date&submit=Submit), [M. Shigemori](http://clincancerres.aacrjournals.org/search?author1=Minoru+Shigemori&sortspec=date&submit=Submit), [Kyogo Itoh](http://clincancerres.aacrjournals.org/search?author1=Kyogo+Itoh&sortspec=date&submit=Submit) and [Ryuichi Tanaka](http://clincancerres.aacrjournals.org/search?author1=Ryuichi+Tanaka&sortspec=date&submit=Submit). 2005. Immunologic evaluation of personalized peptide vaccination for patients with advanced malignant glioma. Clin. Cancer Res. 11:5900-5911. GBM cells and GBM tissue expresses Art-4 by polymerase chain reactions. |
| ***CCNB1*** | *Cyclin B1* | Gliomas express Cyclin B1 during cell cycle: S. Kakino, K. Sasaki, A. Kurose, H. Ito. Intracellular localization of cyclin B1 during the cell cycle in glioma cells. Cytometry 24:49-54, 1996. CTLs were found in patients with breast cancer. R. Bæk, R.S. Andersen, I.M. Svane, L. Engell-Noerregaard, S.R. Hadrup, E. Balslev, M.H. Andersen, and P.T. Straten. CD8 T-cell responses against Cyclin B1 in breast cancer patients with tumors. Overexpressing p53 Clin. Cancer Res. 2009 15:1543-1549. |
| ***EPHA2*** | *EPH receptor A2 (ephrin type-A receptor 2)* | Surgically resected adult and pediatric tissue expressed this antigen mRNA. Zhang JG, E. Newcomb, D. Zagzag, C. Kruse, L. Driggers, N. Hoa, M.R. Jadus. 2008. Tumor antigenic profiles of adult and pediatric brain tumors. J. Neuro-Oncol. 88:65-76.Expression of this molecule within human brain cancers at the protein level: [J. Wykosky](http://clincancerres.aacrjournals.org/search?author1=Jill+Wykosky&sortspec=date&submit=Submit), [D.M. Gibo](http://clincancerres.aacrjournals.org/search?author1=Denise+M.+Gibo&sortspec=date&submit=Submit), [C. Stanton](http://clincancerres.aacrjournals.org/search?author1=Constance+Stanton&sortspec=date&submit=Submit) and [W. Debinski](http://clincancerres.aacrjournals.org/search?author1=Waldemar+Debinski&sortspec=date&submit=Submit). Interleukin-13 Receptor α2, EphA2, and Fos-Related Antigen 1 as Molecular Denominators of High-Grade Astrocytomas and Specific Targets for Combinatorial Therapy. Clin Cancer Res January 1, 2008 14; 199-208. **Induction of CD8+ T-cell responses against novel glioma-associated antigen peptides and clinical activity by vaccination with a-Type 1 polarized dendritic cells and polyinosinic-polycytidylic acid stabilized by lysine and carboxymethylcellulose in patients with recurrent malignant glioma. J. Clin. Oncol. 29:330-336. Lymphocytes from a DC-based clinical trial showed they responded to antigen-pulsed DC produced CD8+ cells that responded by ELISPOT towards EphA2 peptide.** |
| ***EZH2*** | *enhancer of zeste homolog 2 (Drosophila)* | Histone-lysine N-methyltransferase EZH2 is up-regulated in gliomas and GBM stem-like cells: F. Orzan, S. Pellegatta, P. L. Poliani, F. Pisati, V. Caldera, F. Menghi, D. Kapetis, C. Marras, D. Schiffer, G. Finocchiaro. Enhancer of Zeste 2 (EZH2) is up-regulated in malignant gliomas and in glioma stem-like cells. Neuropathology and Applied Neurobiology 37:381-94, 2011. CTLs against HLA-A2 restricted EZH2 epitopes among various cancers but they did not test gliomas. J.C. Steele, E.E. Torr, K.L. Noakes, E. Kalk, P.A. Moss, G.M. Reynolds, S. G. Hubscher, M. van Lohuizen, D.H. Adams, and L.S. Young. 2006. The polycomb group proteins, BMI-1 and EZH2, are tumor-associated antigens. British Journal of Cancer 95, 1202–1211. But Yajima, et al., showed EZH2 expression within GBM cells and GBM tissue. [N. Yajima](http://clincancerres.aacrjournals.org/search?author1=Naoki+Yajima&sortspec=date&submit=Submit), [R. Yamanaka](http://clincancerres.aacrjournals.org/search?author1=Ryuya+Yamanaka&sortspec=date&submit=Submit), [T. Mine](http://clincancerres.aacrjournals.org/search?author1=Takashi+Mine&sortspec=date&submit=Submit), [N. Tsuchiya](http://clincancerres.aacrjournals.org/search?author1=Naoto+Tsuchiya&sortspec=date&submit=Submit), [J. Homma](http://clincancerres.aacrjournals.org/search?author1=Jumpei+Homma&sortspec=date&submit=Submit), [M. Sano](http://clincancerres.aacrjournals.org/search?author1=Masakazu+Sano&sortspec=date&submit=Submit), [T. Kuramoto](http://clincancerres.aacrjournals.org/search?author1=Terukazu+Kuramoto&sortspec=date&submit=Submit), [Y. Obata](http://clincancerres.aacrjournals.org/search?author1=Yayoi+Obata&sortspec=date&submit=Submit), [N. Komatsu](http://clincancerres.aacrjournals.org/search?author1=Nobukazu+Komatsu&sortspec=date&submit=Submit), [Y. Arima](http://clincancerres.aacrjournals.org/search?author1=Yoshimi+Arima&sortspec=date&submit=Submit), [A.Yamada](http://clincancerres.aacrjournals.org/search?author1=Akira+Yamada&sortspec=date&submit=Submit), [M. Shigemori](http://clincancerres.aacrjournals.org/search?author1=Minoru+Shigemori&sortspec=date&submit=Submit), [K. Itoh](http://clincancerres.aacrjournals.org/search?author1=Kyogo+Itoh&sortspec=date&submit=Submit). and [R. Tanaka](http://clincancerres.aacrjournals.org/search?author1=Ryuichi+Tanaka&sortspec=date&submit=Submit). 2005. Immunologic evaluation of personalized peptide vaccination for patients with advanced malignant glioma. Clin. Cancer Res.: 11:5900-5911. Surgically resected adult and pediatric tissue expressed this antigen mRNA. Zhang JG, E. Newcomb, D. Zagzag, C. Kruse, L. Driggers, N. Hoa, M.R. Jadus. 2008. Tumor antigenic profiles of adult and pediatric brain tumors. J. Neuro-Oncol. 88:65-76.CTLs were developed against EZH2 peptides that killed a variety of human prostate cancers: [Itoh Y](http://www.ncbi.nlm.nih.gov/pubmed?term=%22Itoh%20Y%22%5BAuthor%5D), [Komohara Y](http://www.ncbi.nlm.nih.gov/pubmed?term=%22Komohara%20Y%22%5BAuthor%5D), [Komatsu N](http://www.ncbi.nlm.nih.gov/pubmed?term=%22Komatsu%20N%22%5BAuthor%5D), [Minami T](http://www.ncbi.nlm.nih.gov/pubmed?term=%22Minami%20T%22%5BAuthor%5D), [Saito K](http://www.ncbi.nlm.nih.gov/pubmed?term=%22Saito%20K%22%5BAuthor%5D), [Noguchi M](http://www.ncbi.nlm.nih.gov/pubmed?term=%22Noguchi%20M%22%5BAuthor%5D), [Itoh K](http://www.ncbi.nlm.nih.gov/pubmed?term=%22Itoh%20K%22%5BAuthor%5D), [Harada M](http://www.ncbi.nlm.nih.gov/pubmed?term=%22Harada%20M%22%5BAuthor%5D). New peptides of the polycomb group protein enhancer of zeste homolog 2 with the potential to induce cancer-reactive cytotoxic T lymphocytes in human leukocyte antigen-A2+ prostate cancer patients. [Oncol Rep.](http://www.ncbi.nlm.nih.gov/pubmed/17914578) 2007, 18:1231-7. Ogata R, Matsueda S, Yao A, Noguchi M, Itoh K, Harada M. Identification of polycomb group protein enhancer of zeste homolog 2 (EZH2)-derived peptides immunogenic in HLA-A24+ prostate cancer patients. Prostate 2004; 60: 273-81. |
| ***Fosl1*** | *Fos-like antigen-1* | Also known as FRA-1 (Fos-Related antigen-1): Surgically resected adult and pediatric tissue can express this antigen by qRT-PCR. Zhang JG, E. Newcomb, D. Zagzag, C. Kruse, L. Driggers, N. Hoa, M.R. Jadus. 2008. Tumor antigenic profiles of adult and pediatric brain tumors. J. Neuro-Oncol. 88:65-76. [W. Debinski](http://mcr.aacrjournals.org/search?author1=Waldemar+Debinski&sortspec=date&submit=Submit) and [D.M. Gibo](http://mcr.aacrjournals.org/search?author1=Denise+M.+Gibo&sortspec=date&submit=Submit). 2005. Fos-Related Antigen 1 modulates malignant features of glioma cells. Mol. Cancer Res. 3: 237-244. This paper shows the expression of this protein antigen within GBM cells and GBM tissue. **M.R. Young and N.H. Colburn.** 2006. Fra-1 a target for cancer prevention or intervention. [Gene](http://www.sciencedirect.com/science/journal/03781119) [379](http://www.sciencedirect.com/science?_ob=PublicationURL&_tockey=%23TOC%234941%232006%23996209999%23630628%23FLA%23&_cdi=4941&_pubType=J&view=c&_auth=y&_acct=C000050221&_version=1&_urlVersion=0&_userid=10&md5=3cb4bbab30c833c0c7258b8681d34f85): 1-11. This paper shows the versatility of CTLs against various tumor cells that express this antigen. [N. Yajima](http://clincancerres.aacrjournals.org/search?author1=Naoki+Yajima&sortspec=date&submit=Submit), [R. Yamanaka](http://clincancerres.aacrjournals.org/search?author1=Ryuya+Yamanaka&sortspec=date&submit=Submit), [T. Mine](http://clincancerres.aacrjournals.org/search?author1=Takashi+Mine&sortspec=date&submit=Submit), [N. Tsuchiya](http://clincancerres.aacrjournals.org/search?author1=Naoto+Tsuchiya&sortspec=date&submit=Submit), [J. Homma](http://clincancerres.aacrjournals.org/search?author1=Jumpei+Homma&sortspec=date&submit=Submit), [M. Sano](http://clincancerres.aacrjournals.org/search?author1=Masakazu+Sano&sortspec=date&submit=Submit), [T. Kuramoto](http://clincancerres.aacrjournals.org/search?author1=Terukazu+Kuramoto&sortspec=date&submit=Submit), [Y. Obata](http://clincancerres.aacrjournals.org/search?author1=Yayoi+Obata&sortspec=date&submit=Submit), [N. Komatsu](http://clincancerres.aacrjournals.org/search?author1=Nobukazu+Komatsu&sortspec=date&submit=Submit), [Y. Arima](http://clincancerres.aacrjournals.org/search?author1=Yoshimi+Arima&sortspec=date&submit=Submit), [A.Yamada](http://clincancerres.aacrjournals.org/search?author1=Akira+Yamada&sortspec=date&submit=Submit), [M. Shigemori](http://clincancerres.aacrjournals.org/search?author1=Minoru+Shigemori&sortspec=date&submit=Submit), [K. Itoh](http://clincancerres.aacrjournals.org/search?author1=Kyogo+Itoh&sortspec=date&submit=Submit). and [R. Tanaka](http://clincancerres.aacrjournals.org/search?author1=Ryuichi+Tanaka&sortspec=date&submit=Submit). 2005. Immunologic evaluation of personalized peptide vaccination for patients with advanced malignant glioma. Clin. Cancer Res. 2005. 11:5900-5911. |
| ***GnT-V*** | *N-acetyl-glucosaminyl-transferase V* | Gliomas express this glycan: [H. Yamamoto](http://cancerres.aacrjournals.org/search?author1=Hirotaka+Yamamoto&sortspec=date&submit=Submit), [J. Swoger](http://cancerres.aacrjournals.org/search?author1=Jason+Swoger&sortspec=date&submit=Submit), [S. Greene](http://cancerres.aacrjournals.org/search?author1=Suzanne+Greene&sortspec=date&submit=Submit), [T. Saito](http://cancerres.aacrjournals.org/search?author1=Tasuku+Saito&sortspec=date&submit=Submit), [J. Hurh](http://cancerres.aacrjournals.org/search?author1=Jay+Hurh&sortspec=date&submit=Submit), [C. Sweeley](http://cancerres.aacrjournals.org/search?author1=Charla+Sweeley&sortspec=date&submit=Submit), [J. Leestma](http://cancerres.aacrjournals.org/search?author1=Jan+Leestma&sortspec=date&submit=Submit), [E. Mkrdichian](http://cancerres.aacrjournals.org/search?author1=Edward+Mkrdichian&sortspec=date&submit=Submit), [L. Cerullo](http://cancerres.aacrjournals.org/search?author1=Leonard+Cerullo&sortspec=date&submit=Submit), [A. Nishikawa](http://cancerres.aacrjournals.org/search?author1=Atsushi+Nishikawa&sortspec=date&submit=Submit), [Y. Ihara](http://cancerres.aacrjournals.org/search?author1=Yoshito+Ihara&sortspec=date&submit=Submit), [N. Taniguchi](http://cancerres.aacrjournals.org/search?author1=Naoyuki+Taniguchi&sortspec=date&submit=Submit), and [J.Moskal](http://cancerres.aacrjournals.org/search?author1=Joseph+R.+Moskal&sortspec=date&submit=Submit). β1,6-N-Acetylglucosamine-bearing N-Glycans in Human Gliomas: Implications for a Role in Regulating Invasivity. Cancer Res 60:134, 2000. CTLs recognize GnT-V peptides on melanomas: [Y Guilloux](http://jem.rupress.org/search?author1=Y+Guilloux&sortspec=date&submit=Submit), [S Lucas](http://jem.rupress.org/search?author1=S+Lucas&sortspec=date&submit=Submit), [V G Brichard](http://jem.rupress.org/search?author1=V+G+Brichard&sortspec=date&submit=Submit), [A Van Pel](http://jem.rupress.org/search?author1=A+Van+Pel&sortspec=date&submit=Submit), [C Viret](http://jem.rupress.org/search?author1=C+Viret&sortspec=date&submit=Submit), [E De Plaen](http://jem.rupress.org/search?author1=E+De+Plaen&sortspec=date&submit=Submit), [F Brasseur](http://jem.rupress.org/search?author1=F+Brasseur&sortspec=date&submit=Submit), [B Lethé](http://jem.rupress.org/search?author1=B+Leth%C3%A9&sortspec=date&submit=Submit), [F Jotereau](http://jem.rupress.org/search?author1=F+Jotereau&sortspec=date&submit=Submit), and T Boon A peptide recognized by human cytolytic T lymphocytes on HLA-A2 melanomas is encoded by an intron sequence of the N-acetylglucosaminyltransferase V gene 1996 JEM 183 : 1173-1183. |
| ***GP-100*** | *Glycoprotein 100* | U87 and U118 glioma cells were positive by RT-PCR; 38% of fresh human GBM were positive for this mRNA. D. D. Chi, R. E. Merchant, R. Rand, A. J. Conrad, D. Garrison, R. Turner, D. L. Morton, and D. S. Hoon. Molecular detection of tumor-associated antigens shared by human cutaneous melanomas and gliomas. Am J Pathol. 1997 150: 2143–52. This antigen is expressed within gliomas and CTL responses can be seen: [G. Liu](http://cancerres.aacrjournals.org/search?author1=Gentao+Liu&sortspec=date&submit=Submit), [H. Ying](http://cancerres.aacrjournals.org/search?author1=Han+Ying&sortspec=date&submit=Submit), [G. Zeng](http://cancerres.aacrjournals.org/search?author1=Gang+Zeng&sortspec=date&submit=Submit), [C.J. Wheeler](http://cancerres.aacrjournals.org/search?author1=Christopher+J.+Wheeler&sortspec=date&submit=Submit), [K.L. Black](http://cancerres.aacrjournals.org/search?author1=Keith+L.+Black&sortspec=date&submit=Submit), and [J.S. Yu](http://cancerres.aacrjournals.org/search?author1=John+S.+Yu&sortspec=date&submit=Submit). HER-2, gp100, and MAGE-1 Are Expressed in Human Glioblastoma and Recognized by Cytotoxic T Cells. Cancer Res. 64: 4980-6. 2004. |
| ***Her2*** | *Human Epidermal growth factor Receptor 2* | Also known as proto-oncogene Neu, receptor tyrosine-protein kinase erbB-2, CD340. This antigen is expressed within gliomas and CTL responses can be seen: [G. Liu](http://cancerres.aacrjournals.org/search?author1=Gentao+Liu&sortspec=date&submit=Submit), [H. Ying](http://cancerres.aacrjournals.org/search?author1=Han+Ying&sortspec=date&submit=Submit), [G. Zeng](http://cancerres.aacrjournals.org/search?author1=Gang+Zeng&sortspec=date&submit=Submit), [C.J. Wheeler](http://cancerres.aacrjournals.org/search?author1=Christopher+J.+Wheeler&sortspec=date&submit=Submit), [K.L. Black](http://cancerres.aacrjournals.org/search?author1=Keith+L.+Black&sortspec=date&submit=Submit), and [J.S. Yu](http://cancerres.aacrjournals.org/search?author1=John+S.+Yu&sortspec=date&submit=Submit). HER-2, gp100, and MAGE-1 Are Expressed in Human Glioblastoma and Recognized by Cytotoxic T Cells. Cancer Res. 64: 4980-6. 2004. Also chimeric receptor redirected T cells are being developed as a monotherapy: [Ahmed, N](http://www.ncbi.nlm.nih.gov/pubmed?term=%22Ahmed%20N%22%5BAuthor%5D&itool=EntrezSystem2.PEntrez.Pubmed.Pubmed_ResultsPanel.Pubmed_RVAbstract)., V.S. Salsman, Y. Kew, D. Shaffer, S. Powell, Y.J. Zhang, R.G. Grossman, H.E. Heslop, and S. Gottschalk. 2010. **HER2-specific T cells target primary glioblastoma stem cells and induce regression of autologous experimental tumors.** Clin. [Cancer Res.](javascript:AL_get(this,%20'jour',%20'Cancer%20Res.');) 16:474-85 |
| ***HNRPL*** | *Heterogeneous nuclear ribonucleoprotein L* | Gene expressed at mRNA level and CTLs could be developed and could react with human glioma cells: [N. Yajima](http://clincancerres.aacrjournals.org/search?author1=Naoki+Yajima&sortspec=date&submit=Submit), [R. Yamanaka](http://clincancerres.aacrjournals.org/search?author1=Ryuya+Yamanaka&sortspec=date&submit=Submit), [T. Mine](http://clincancerres.aacrjournals.org/search?author1=Takashi+Mine&sortspec=date&submit=Submit), [N. Tsuchiya](http://clincancerres.aacrjournals.org/search?author1=Naoto+Tsuchiya&sortspec=date&submit=Submit), [J. Homma](http://clincancerres.aacrjournals.org/search?author1=Jumpei+Homma&sortspec=date&submit=Submit), [M. Sano](http://clincancerres.aacrjournals.org/search?author1=Masakazu+Sano&sortspec=date&submit=Submit), [T. Kuramoto](http://clincancerres.aacrjournals.org/search?author1=Terukazu+Kuramoto&sortspec=date&submit=Submit), [Y. Obata](http://clincancerres.aacrjournals.org/search?author1=Yayoi+Obata&sortspec=date&submit=Submit), [N. Komatsu](http://clincancerres.aacrjournals.org/search?author1=Nobukazu+Komatsu&sortspec=date&submit=Submit), [Y. Arima](http://clincancerres.aacrjournals.org/search?author1=Yoshimi+Arima&sortspec=date&submit=Submit), [A. Yamada](http://clincancerres.aacrjournals.org/search?author1=Akira+Yamada&sortspec=date&submit=Submit), [M. Shigemori](http://clincancerres.aacrjournals.org/search?author1=Minoru+Shigemori&sortspec=date&submit=Submit), [K. Itoh](http://clincancerres.aacrjournals.org/search?author1=Kyogo+Itoh&sortspec=date&submit=Submit) and [R. Tanaka](http://clincancerres.aacrjournals.org/search?author1=Ryuichi+Tanaka&sortspec=date&submit=Submit). Immunologic Evaluation of Personalized Peptide Vaccination for Patients with Advanced Malignant Glioma. Clin Cancer Res August 15, 2005 11; 5900. CD8+ T cells release IFN-γ in response to pancreatic tumor cells: [M. Ito](http://cancerres.aacrjournals.org/search?author1=Masaaki+Ito&sortspec=date&submit=Submit), [S. Shichijo](http://cancerres.aacrjournals.org/search?author1=Shigeki+Shichijo&sortspec=date&submit=Submit), [N. Tsuda](http://cancerres.aacrjournals.org/search?author1=Naotake+Tsuda&sortspec=date&submit=Submit), [M. Ochi](http://cancerres.aacrjournals.org/search?author1=Mika+Ochi&sortspec=date&submit=Submit), [N. Harashima](http://cancerres.aacrjournals.org/search?author1=Nanae+Harashima&sortspec=date&submit=Submit), [N. Saito](http://cancerres.aacrjournals.org/search?author1=Norio+Saito&sortspec=date&submit=Submit), and [K. Itoh](http://cancerres.aacrjournals.org/search?author1=Kyogo+Itoh&sortspec=date&submit=Submit). Molecular Basis of T Cell-mediated Recognition of Pancreatic Cancer Cells. Cancer Res March 3, 2001 61; 2038-46. Surgically resected adult and pediatric tissue expressed this antigen mRNA. Zhang JG, E. Newcomb, D. Zagzag, C. Kruse, L. Driggers, N. Hoa, M.R. Jadus. 2008. Tumor antigenic profiles of adult and pediatric brain tumors. J. Neuro-Oncol. 88:65-76. |
| ***IL13Rα2*** | *Interleukin-13 receptor subunit alpha-2* | Expression of this molecule within human brain cancers: [J. Wykosky](http://clincancerres.aacrjournals.org/search?author1=Jill+Wykosky&sortspec=date&submit=Submit), [D.M. Gibo](http://clincancerres.aacrjournals.org/search?author1=Denise+M.+Gibo&sortspec=date&submit=Submit), [C. Stanton](http://clincancerres.aacrjournals.org/search?author1=Constance+Stanton&sortspec=date&submit=Submit) and [W. Debinski](http://clincancerres.aacrjournals.org/search?author1=Waldemar+Debinski&sortspec=date&submit=Submit). Interleukin-13 Receptor α2, EphA2, and Fos-Related Antigen 1 as Molecular Denominators of High-Grade Astrocytomas and Specific Targets for Combinatorial Therapy. Clin Cancer Res January 1, 2008 14; 199-208. Chimeric receptor redirected T cells exist for this antigen in human gliomas: K. S. Kahlon, C. Brown, L. J. N. Cooper, A. Raubitschek, S. J. Forman, and M. C. Jensen, “Specific recognition and killing of glioblastomamultiforme by interleukin 13-zetakine redirected cytolytic T cells,” Cancer Research, 64: 9160–9166, 2004. |
| ***MageA1*** | *Melanoma-associated antigen A1* | U87 and U118 glioma cells were positive by RT-PCR; 38% of fresh human GBM were positive for this mRNA. D. D. Chi, R. E. Merchant, R. Rand, A. J. Conrad, D. Garrison, R. Turner, D. L. Morton, and D. S. Hoon. Molecular detection of tumor-associated antigens shared by human cutaneous melanomas and gliomas. Am J Pathol. 1997 150: 2143–52. This antigen is expressed within gliomas and CTL responses can be seen: [G. Liu](http://cancerres.aacrjournals.org/search?author1=Gentao+Liu&sortspec=date&submit=Submit), [H. Ying](http://cancerres.aacrjournals.org/search?author1=Han+Ying&sortspec=date&submit=Submit), [G. Zeng](http://cancerres.aacrjournals.org/search?author1=Gang+Zeng&sortspec=date&submit=Submit), [C.J. Wheeler](http://cancerres.aacrjournals.org/search?author1=Christopher+J.+Wheeler&sortspec=date&submit=Submit), [K.L. Black](http://cancerres.aacrjournals.org/search?author1=Keith+L.+Black&sortspec=date&submit=Submit), and [J.S. Yu](http://cancerres.aacrjournals.org/search?author1=John+S.+Yu&sortspec=date&submit=Submit). HER-2, gp100, and MAGE-1 Are Expressed in Human Glioblastoma and Recognized by Cytotoxic T Cells. Cancer Res. 64: 4980-6. 2004. |
| ***MRP-3*** | *Multidrug Resistance-associated Protein -3* | Expression was found to be higher in GBM stem like cells: *Andrea Salmaggi, Amerigo Boiardi, Maurizio Gelati, Annamaria Russo, Chiara Calatozzolo, Emilio Ciusani, Francesca Luisa Sciacca, Arianna Ottolina, Eugenio Gostino Parati, Caterina La Porta, Giulio Alessandri, Carlo Marras, Danilo Croci, Marco De Rossi. Glioblastoma-derived tumorospheres identify a population of tumor stem-like cells with angiogenic potential and enhanced multidrug resistance phenotype. Glia* [*54*](http://onlinelibrary.wiley.com/doi/10.1002/glia.v54:8/issuetoc)*: 850–60, 2006. And :Kuan CT, Wakiya K, Herndon JE, Lipp ES, Pegram CN, Riggins GJ, Rasheed A, Szafranski SE, McLendon RE, Wikstrand CJ, Bigner DD: MRP3: a molecular target for human glioblastoma multiforme immunotherapy. BMC Cancer 2010, 10:468. Single chain antibodies being developed against human gliomas: Kuan CT, Srivastava N, McLendon RE, Marasco WA, Zalutsky MR, Bigner DD: Recombinant single-chain variable fragment antibodies against extracellular epitopes of human multidrug resistance protein MRP3 fortargeting malignant gliomas. Int J Cancer 2010, 127(3):598-611.*  ***CTLs vs MRP-3 were developed:  Y. Komohara, M. Harada, Y. Arima, S. Suekane, M. Noguchi, A. Yamada, K. Itoh, K. Matsuoka.*** *Identification of Target Antigens in Specific Immunotherapy for Renal Cell Carcinoma* [*Journal of Urology*](http://www.sciencedirect.com/science/journal/00225347)  [*177*](http://www.sciencedirect.com/science?_ob=PublicationURL&_hubEid=1-s2.0-S0022534707X0020X&_cid=273470&_pubType=JL&view=c&_auth=y&_acct=C000055380&_version=1&_urlVersion=0&_userid=1928909&md5=3423ecc42bc26ad7be1d5cb27774cdc2)*: 1157-1162, 2007. CTLs were developed against these MRP-3 peptides:* [*A.Yamada*](http://cancerres.aacrjournals.org/search?author1=Akira+Yamada&sortspec=date&submit=Submit)*,* [*K. Kawano*](http://cancerres.aacrjournals.org/search?author1=Kouichiro+Kawano&sortspec=date&submit=Submit)*,* [*M. Koga*](http://cancerres.aacrjournals.org/search?author1=Makoto+Koga&sortspec=date&submit=Submit)*,* [*T. Matsumoto*](http://cancerres.aacrjournals.org/search?author1=Tomoko+Matsumoto&sortspec=date&submit=Submit)*, and* [*K. Itoh*](http://cancerres.aacrjournals.org/search?author1=Kyogo+Itoh&sortspec=date&submit=Submit)*. Multidrug Resistance-associated Protein 3 Is a Tumor Rejection Antigen Recognized by HLA-A2402-restricted Cytotoxic T Lymphocytes Cancer Res. 2001* 61: *6459-66. Clinical trials are being used for this antigen: M. Terasaki, S. Shibui, Y. Narita, T. Fujimaki, T. Aoki, K. Kajiwara,Y. Sawamura, K. Kurisu, T. Mineta, A. Yamada, and K. Itoh. Phase I Trial of a Personalized Peptide Vaccine for Patients Positive for Human Leukocyte Antigen-A24 With Recurrent or Progressive Glioblastoma Multiforme. JCO 2011 29:337-344.* |
| ***PTH-rP*** | *Parathyroid Hormone-related Peptide* | This hormone-like molecule was found to be made by human gliomas: F.S. Pardo, W.W. Lien, H.S. Fox, J.T. Efird, J.A. Aguilera. D.W. Burton, L. J. Deftos. 2004. Parathyroid hormone–related protein expression is correlated with clinical course in patients with glial tumors. Cancer 101:2622-28.  Others have shown that CTL epitopes are present against this peptide: 1) [N. Yajima](http://clincancerres.aacrjournals.org/search?author1=Naoki+Yajima&sortspec=date&submit=Submit), [R. Yamanaka](http://clincancerres.aacrjournals.org/search?author1=Ryuya+Yamanaka&sortspec=date&submit=Submit), [T. Mine](http://clincancerres.aacrjournals.org/search?author1=Takashi+Mine&sortspec=date&submit=Submit), [N. Tsuchiya](http://clincancerres.aacrjournals.org/search?author1=Naoto+Tsuchiya&sortspec=date&submit=Submit), [J. Homma](http://clincancerres.aacrjournals.org/search?author1=Jumpei+Homma&sortspec=date&submit=Submit), [M. Sano](http://clincancerres.aacrjournals.org/search?author1=Masakazu+Sano&sortspec=date&submit=Submit), [T. Kuramoto](http://clincancerres.aacrjournals.org/search?author1=Terukazu+Kuramoto&sortspec=date&submit=Submit), [Y. Obata](http://clincancerres.aacrjournals.org/search?author1=Yayoi+Obata&sortspec=date&submit=Submit), [N. Komatsu](http://clincancerres.aacrjournals.org/search?author1=Nobukazu+Komatsu&sortspec=date&submit=Submit), [Y. Arima](http://clincancerres.aacrjournals.org/search?author1=Yoshimi+Arima&sortspec=date&submit=Submit), [A.Yamada](http://clincancerres.aacrjournals.org/search?author1=Akira+Yamada&sortspec=date&submit=Submit), [M. Shigemori](http://clincancerres.aacrjournals.org/search?author1=Minoru+Shigemori&sortspec=date&submit=Submit), [K. Itoh](http://clincancerres.aacrjournals.org/search?author1=Kyogo+Itoh&sortspec=date&submit=Submit). and [R. Tanaka](http://clincancerres.aacrjournals.org/search?author1=Ryuichi+Tanaka&sortspec=date&submit=Submit). 2005. Immunologic evaluation of personalized peptide vaccination for patients with advanced malignant glioma. Clin Cancer Res.:11:5900-5911. 2) Y. [Arima](http://ukpmc.ac.uk/search/;jsessionid=17617362FF0D176E34D4CF10C54A6044.jvm1?page=1&query=AUTH:%22Arima+Y%22+SORT_DATE:y), S. Find all citations by this author (default).  Or [filter your current search](http://ukpmc.ac.uk/search/;jsessionid=17617362FF0D176E34D4CF10C54A6044.jvm1?page=1&query=arima,+y,+s.+matsueda+%26+parathyroid+AND+AUTH:%22Matsueda+S%22&refineauth=Matsueda+S)  [Matsueda](http://ukpmc.ac.uk/search/;jsessionid=17617362FF0D176E34D4CF10C54A6044.jvm1?page=1&query=AUTH:%22Matsueda+S%22+SORT_DATE:y), H. Find all citations by this author (default).  Or [filter your current search](http://ukpmc.ac.uk/search/;jsessionid=17617362FF0D176E34D4CF10C54A6044.jvm1?page=1&query=arima,+y,+s.+matsueda+%26+parathyroid+AND+AUTH:%22Yano+H%22&refineauth=Yano+H)  [Yano](http://ukpmc.ac.uk/search/;jsessionid=17617362FF0D176E34D4CF10C54A6044.jvm1?page=1&query=AUTH:%22Yano+H%22+SORT_DATE:y), M. Find all citations by this author (default).  Or [filter your current search](http://ukpmc.ac.uk/search/;jsessionid=17617362FF0D176E34D4CF10C54A6044.jvm1?page=1&query=arima,+y,+s.+matsueda+%26+parathyroid+AND+AUTH:%22Harada+M%22&refineauth=Harada+M)  [Harada](http://ukpmc.ac.uk/search/;jsessionid=17617362FF0D176E34D4CF10C54A6044.jvm1?page=1&query=AUTH:%22Harada+M%22+SORT_DATE:y), and Find all citations by this author (default).  Or [filter your current search](http://ukpmc.ac.uk/search/;jsessionid=17617362FF0D176E34D4CF10C54A6044.jvm1?page=1&query=arima,+y,+s.+matsueda+%26+parathyroid+AND+AUTH:%22Itoh+K%22&refineauth=Itoh+K) [Itoh K](http://ukpmc.ac.uk/search/;jsessionid=17617362FF0D176E34D4CF10C54A6044.jvm1?page=1&query=AUTH:%22Itoh+K%22+SORT_DATE:y). 2005. Parathyroid hormone-related protein as a common target molecule in specific immunotherapy for a wide variety of tumor types. Internatl. J. Oncol. 27:981-988. This group produced HLA-A24 restricted CTLs against various cancer cells. 3) G. Francini, A. Scardino, K. Kosmatopoulos, F.A. Lemonnier, G. Campoccia, M. Sabatino, D. Pozzessere, R. Petrioli, L. Lozzi, P. Neri, G. Fanetti, M. G. Cusi, and P. Correale. 2002. High-Affinity HLA-A02.01 peptides from parathyroid hormone-related protein generate in vitro and in vivo antitumor CTL response without autoimmune side effects. J Immunol. 169: 4840-4849. CTLs killed HLA-A2+ breast and prostate cancers bearing PTH-rP.Surgically resected adult and pediatric tissue express this antigen. Zhang JG, E. Newcomb, D. Zagzag, C. Kruse, L. Driggers, N. Hoa, M.R. Jadus. 2008. Tumor antigenic profiles of adult and pediatric brain tumors. J. Neuro-Oncol. 88:65-76. |
| ***PRAME*** | *Preferentially expressed Antigen in Mlanoma* | Initially, this tumor antigen was found within melanoma cells. The transcript is an open reading frame and comes from 2 spliced variant forms. H. Mamoru; Y. Li, M. El-Gamil, G.A. Ohnmacht, S.A. Rosenberg, and P.F. Robbins. 2001. Melanoma-Reactive CD8 + T Cells Recognize a Novel Tumor Antigen Expressed in a Wide Variety of Tumor Types. J .Immunother 24: 323-333. Since melanomas and glioma share a common progeny cell. This antigen was subsequently discovered to be expressed within glioma cells. HLA-A1 restricted and CTLs were able to recognize this antigen when it is expressed within human gliomas. G. Liu, J.S. Yu, G. Zeng, D, Yin, D. Xie, K.L. Black, and H. Ying. 2004. AIM-2: A novel tumor antigen is expressed and presented by human glioma cells. J. Immunotherapy: 2004, 27: 220-226. Surgically resected adult and pediatric tissue can express this antigen at the mRNA level. Zhang JG, E. Newcomb, D. Zagzag, C. Kruse, L. Driggers, N. Hoa, M.R. Jadus. 2008. Tumor antigenic profiles of adult and pediatric brain tumors. J. Neuro-Oncol. 88:65-76. |
| ***SART1*** | *Squamous cell carcinoma Antigen Recognized by T cells* | Surgically resected adult and pediatric tissue can express this antigen. Zhang JG, E. Newcomb, D. Zagzag, C. Kruse, L. Driggers, N. Hoa, M.R. Jadus. 2008. Tumor antigenic profiles of adult and pediatric brain tumors. J. Neuro-Oncol. 88:65-76. CTLs were developed for this antigen: [S. Shichijo](http://jem.highwire.org/search?author1=Shigeki+Shichijo&sortspec=date&submit=Submit), [M. Nakao](http://jem.highwire.org/search?author1=Masanobu+Nakao&sortspec=date&submit=Submit), [Y. Imai](http://jem.highwire.org/search?author1=Yasuhisa+Imai&sortspec=date&submit=Submit), [H. Takasu](http://jem.highwire.org/search?author1=Hideo+Takasu&sortspec=date&submit=Submit), [M. Kawamoto](http://jem.highwire.org/search?author1=Mayumi+Kawamoto&sortspec=date&submit=Submit), [F. Niiya](http://jem.highwire.org/search?author1=Fumihiko+Niiya&sortspec=date&submit=Submit), [D. Yang](http://jem.highwire.org/search?author1=Damu+Yang&sortspec=date&submit=Submit), [Y. Toh](http://jem.highwire.org/search?author1=Yuji+Toh&sortspec=date&submit=Submit), [H. Yamana](http://jem.highwire.org/search?author1=Hideaki+Yamana&sortspec=date&submit=Submit), and [K. Itoh](http://jem.highwire.org/search?author1=Kyogo+Itoh&sortspec=date&submit=Submit) A Gene Encoding Antigenic Peptides of Human Squamous Cell Carcinoma Recognized by Cytotoxic T Lymphocytes. J Exp. Med. 187:277-288, 1998. CTLs towards Sart-1 will kill human gliomas: T. Imaizumi, T. Kuramoto, K. Matsunaga, S. Shichijo, S. Yutani, M. Shigemori, K. Oizumi, Kyogo Itoh. Expression of the tumor-rejection antigen SART1 in brain tumors International Journal of Cancer [83:](http://onlinelibrary.wiley.com/doi/10.1002/(SICI)1097-0215(19991210)83:6%3c%3e1.0.CO;2-V/issuetoc) 760–764, 1999. |
| ***SART2*** | *Squamous cell carcinoma Antigen Recognized by T cells 2* | Surgically resected adult and pediatric tissue can express this antigen. Zhang JG, E. Newcomb, D. Zagzag, C. Kruse, L. Driggers, N. Hoa, M.R. Jadus. 2008. Tumor antigenic profiles of adult and pediatric brain tumors. J. Neuro-Oncol. 88:65-76. Sart-2 protein was found in 18 of 18 human GBMs and CTLs were generated against squamous carcinomas: M. Nakao, S. Shichijo, T. Imaizumi, Y. Inoue, K. Matsunaga, A. Yamada, M. Kikuchi, N. Tsuda, K. Ohta, S. Takamori, H. Yamana, H. Fujita and K. Itoh. Identification of a Gene Coding for a New Squamous Cell Carcinoma Antigen Recognized by the CTL Journal of Immunology March 1, 2000 vol. 164 no. 5 2565-2574. |
| ***SART3*** | *Squamous cell carcinoma Antigen Recognized by T-cells 3* | Surgically resected adult and pediatric tissue can express this antigen. Zhang JG, E. Newcomb, D. Zagzag, C. Kruse, L. Driggers, N. Hoa, M.R. Jadus. 2008. Tumor antigenic profiles of adult and pediatric: brain tumors. J. Neuro-Oncol. 88:65-76. CTLs were described against glioma cells within GBM patients: Murayama, K.; Kobayashi, T.; Imaizumi, T.; Matsunaga, K.; Kuramoto, T.; Shigemori, M.;Shichijo, S.; Itoh, K. Expression of the SART3 tumor-rejection antigen in brain tumors and induction of cytotoxic T lymphocytes by its peptides. J. Immunother. 2000, 23, 511-8. |
| **SOX11** | *SRY (sex determining region Y)-box 11* | This is a transcription factor that glioma cells use for protein regulation. [B. Weigle](http://www.cancerletters.info/article/S0304-3835(06)00062-0/abstract##), R. Ebner, A. Temme, S. Schwind, M. Schmitz, A. Kiessling, M.A. Rieger, G. Schakert, H.K. Schackert and P.E. Rieber. 2005. Highly specific over-expression of the transcription factor SOX11 in human malignant gliomas. Oncology Reports 13:139-144. [M. Schmitz](http://www.cancerletters.info/article/S0304-3835(06)00062-0/abstract##), [R. Wehner](http://www.cancerletters.info/article/S0304-3835(06)00062-0/abstract##), [S. Stevanovic](http://www.cancerletters.info/article/S0304-3835(06)00062-0/abstract##), [A. Kiessling](http://www.cancerletters.info/article/S0304-3835(06)00062-0/abstract##), [M.A. Rieger](http://www.cancerletters.info/article/S0304-3835(06)00062-0/abstract##), [A.Temme](http://www.cancerletters.info/article/S0304-3835(06)00062-0/abstract##), [M. Bachmann](http://www.cancerletters.info/article/S0304-3835(06)00062-0/abstract##), [E. P. Rieber](http://www.cancerletters.info/article/S0304-3835(06)00062-0/abstract##), and [B. Weigle](http://www.cancerletters.info/article/S0304-3835(06)00062-0/abstract##). 2007. Identification of a naturally processed T cell epitope derived from the glioma-associated protein SOX11. Cancer Letters [245](http://www.cancerletters.info/issues?Vol=245):331-336. This paper shows a HLA-A2 restricted epitope derived from Sox 11 that allows killing of various glioma cell lines. |
| **Survivin (BIRC5)** | *Baculoviral IAP repeat containing 5* | Also called baculoviral inhibitor of apoptosis repeat-containing 5 or BIRC5. By western blotting the amount of survivin inversely correlated with glioma patient survival. [A. Chakravarti](http://jco.ascopubs.org/search?author1=Arnab+Chakravarti&sortspec=date&submit=Submit), [E. Noll](http://jco.ascopubs.org/search?author1=Elizabeth+Noll&sortspec=date&submit=Submit), [P. McL Black](http://jco.ascopubs.org/search?author1=Peter+McL.+Black&sortspec=date&submit=Submit), [D.F. Finkelstein](http://jco.ascopubs.org/search?author1=Daniel+F.+Finkelstein&sortspec=date&submit=Submit), [D.M. Finkelstein](http://jco.ascopubs.org/search?author1=Dianne+M.+Finkelstein&sortspec=date&submit=Submit), [N.J. Dyson](http://jco.ascopubs.org/search?author1=Nicholas+J.+Dyson&sortspec=date&submit=Submit), [J.S. Loeffler](http://jco.ascopubs.org/search?author1=Jay+S.+Loeffler&sortspec=date&submit=Submit). Quantitatively Determined Survivin Expression Levels Are of Prognostic Value in Human Gliomas J Clin Oncol. 20:1063-8. 2002. CTLs were developed against the survivin peptides. [Y. Hirohashi](http://clincancerres.aacrjournals.org/search?author1=Yoshihiko+Hirohashi&sortspec=date&submit=Submit), [T. Torigoe](http://clincancerres.aacrjournals.org/search?author1=Toshihiko+Torigoe&sortspec=date&submit=Submit), [A. Maeda](http://clincancerres.aacrjournals.org/search?author1=Akiko+Maeda&sortspec=date&submit=Submit), [Y. Nabeta](http://clincancerres.aacrjournals.org/search?author1=Yuki+Nabeta&sortspec=date&submit=Submit), [K. Kamiguchi](http://clincancerres.aacrjournals.org/search?author1=Kenjiro+Kamiguchi&sortspec=date&submit=Submit), [T. Sato](http://clincancerres.aacrjournals.org/search?author1=Takashi+Sato&sortspec=date&submit=Submit), [J. Yoda](http://clincancerres.aacrjournals.org/search?author1=Junichi+Yoda&sortspec=date&submit=Submit), [H. Ikeda](http://clincancerres.aacrjournals.org/search?author1=Hideyuki+Ikeda&sortspec=date&submit=Submit), [K. Hirata](http://clincancerres.aacrjournals.org/search?author1=Kouichi+Hirata&sortspec=date&submit=Submit), [N.Yamanaka](http://clincancerres.aacrjournals.org/search?author1=Noboru+Yamanaka&sortspec=date&submit=Submit), and [N. Sato](http://clincancerres.aacrjournals.org/search?author1=Noriyuki+Sato&sortspec=date&submit=Submit)[.](http://clincancerres.aacrjournals.org/content/8/6/1731.short#fn-2) An HLA-A24-restricted Cytotoxic T Lymphocyte Epitope of a Tumor-associated Protein, Survivin. Clin Cancer res. 8:173-9, 2002. In a mouse model, using survivin pulsed dendritic cells generatet effective immunity towards GL261 glioma cells. [MJ. Ciesielski](http://www.springerlink.com/content/?Author=Michael+J.+Ciesielski), [L Apfel](http://www.springerlink.com/content/?Author=Lisa+Apfel), [TA. Barone](http://www.springerlink.com/content/?Author=Tara+A.+Barone), [CA. Castro](http://www.springerlink.com/content/?Author=Carla+A.+Castro), [TC. Weiss](http://www.springerlink.com/content/?Author=Tina+C.+Weiss) and [R.A. Fenstermaker](http://www.springerlink.com/content/?Author=Robert+A.+Fenstermaker). Antitumor effects of a xenogeneic survivin bone marrow derived dendritic cell vaccine against murine GL261 gliomas [Cancer Immunology, Immunotherapy](http://www.springerlink.com/content/0340-7004/) [Volume 55, Number 12](http://www.springerlink.com/content/0340-7004/55/12/), 1491-1503. Human CTLs against surviving were capable of being generated. EK Kim, HI Cho, SH Yoon, MJ Park, HJ Sohn, HJ Kim, ST Oh, TG Kim. Efficient generation of survivin-specific cytotoxic T lymphocytes from healthy persons in vitro: Quantitative and qualitative effects of CD4+ T cells. [Vaccine](http://www.sciencedirect.com/science/journal/0264410X) [26](http://www.sciencedirect.com/science?_ob=PublicationURL&_hubEid=1-s2.0-S0264410X08X00313&_cid=271205&_pubType=JL&view=c&_auth=y&_acct=C000055380&_version=1&_urlVersion=0&_userid=1928909&md5=a0c89fee27406214f8a8d095d5031a03): 3987-3997, 2008. |
| **TERT** | *TElomerase Reverse Transcriptase* | Gliomas can use Tert to maintain GBM stem like cells: [*Samuel Beck*](http://www.springerlink.com/content/?Author=Samuel+Beck)*,* [*Xun Jin*](http://www.springerlink.com/content/?Author=Xun+Jin)*,* [*Young-Woo Sohn*](http://www.springerlink.com/content/?Author=Young-Woo+Sohn)*,* [*Jun-Kyum Kim*](http://www.springerlink.com/content/?Author=Jun-Kyum+Kim)*,* [*Sung-Hak Kim*](http://www.springerlink.com/content/?Author=Sung-Hak+Kim)*,* [*Jinlong Yin*](http://www.springerlink.com/content/?Author=Jinlong+Yin)*,* [*Xumin Pian*](http://www.springerlink.com/content/?Author=Xumin+Pian)*,* [*Sung-Chan Kim*](http://www.springerlink.com/content/?Author=Sung-Chan+Kim)*,* [*Do-Hyun Nam*](http://www.springerlink.com/content/?Author=Do-Hyun+Nam) *and* [*Yun-Jaie Choi*](http://www.springerlink.com/content/?Author=Yun-Jaie+Choi), et al. *Telomerase activity-independent function of TERT allows glioma cells to attain cancer stem cell characteristics by inducing EGFR expression.* [*Molecules and Cells*](http://www.springerlink.com/content/1016-8478/) [*31*](http://www.springerlink.com/content/1016-8478/31/1/)*: 9-15.* Developed CTLs vs hTert: B. Minev, J Hipp, H. Firat, JD Schmidt, P. Langlade-Demoyen, M. Zanetti. Cytotoxic T cell immuinity against telomerase reverse transcriptase in humans, PNAS, 2000, 97: 4796-801. |
| **Trp-1** | *Tyrosinase-Related Protein 1* | By RT-PCR 52% of fresh human GBM and GBM cell lines were positive for this mRNA. D. D. Chi, R. E. Merchant, R. Rand, A. J. Conrad, D. Garrison, R. Turner, D. L. Morton, and D. S. Hoon. Molecular detection of tumor-associated antigens shared by human cutaneous melanomas and gliomas. Am J Pathol. 1997 150: 2143–52. CTLs developed against Trp-1: Wang, RF, MR Parkhurst, Y Kawakami, PF Robbins and SA Rosenberg. Utilization of an alternative open reading frame of a normal gene in generating a novel human cancer antigen. J. Exp Med. 183:1131-40, 1996. |
| **Trp-2** | *Tyrosinase-Pelated Protein 2* | By RT-PCR GBM cell lines were all positive, while 62% of fresh human GBM were positive for this mRNA. D. D. Chi, R. E. Merchant, R. Rand, A. J. Conrad, D. Garrison, R. Turner, D. L. Morton, and D. S. Hoon. Molecular detection of tumor-associated antigens shared by human cutaneous melanomas and gliomas. Am J Pathol. 1997 150: 2143–52. CTLs versus Trp-2+ glioma also sensitivies them towards various cytotoxic drugs. G. Liu, Y Akasaki, HT Khong, CJ Wheeler, A Das, KL Black and J.S. Yu. Cytotoxic T cell targeting of TRP-2 sensitizes human malignant glioma to chemotherapy. Oncogene (2005) 24, 5226–5234. |
| **Ube2v** | *Ubiquitin-conjugating enzyme E2 variant* | Ube2V gene expressed at mRNA level and CTLs could be developed and could react with human glioma cells: [N. Yajima](http://clincancerres.aacrjournals.org/search?author1=Naoki+Yajima&sortspec=date&submit=Submit), [R. Yamanaka](http://clincancerres.aacrjournals.org/search?author1=Ryuya+Yamanaka&sortspec=date&submit=Submit), [T. Mine](http://clincancerres.aacrjournals.org/search?author1=Takashi+Mine&sortspec=date&submit=Submit), [N. Tsuchiya](http://clincancerres.aacrjournals.org/search?author1=Naoto+Tsuchiya&sortspec=date&submit=Submit), [J. Homma](http://clincancerres.aacrjournals.org/search?author1=Jumpei+Homma&sortspec=date&submit=Submit), [M. Sano](http://clincancerres.aacrjournals.org/search?author1=Masakazu+Sano&sortspec=date&submit=Submit), [T. Kuramoto](http://clincancerres.aacrjournals.org/search?author1=Terukazu+Kuramoto&sortspec=date&submit=Submit), [Y. Obata](http://clincancerres.aacrjournals.org/search?author1=Yayoi+Obata&sortspec=date&submit=Submit), [N. Komatsu](http://clincancerres.aacrjournals.org/search?author1=Nobukazu+Komatsu&sortspec=date&submit=Submit), [Y. Arima](http://clincancerres.aacrjournals.org/search?author1=Yoshimi+Arima&sortspec=date&submit=Submit), [A. Yamada](http://clincancerres.aacrjournals.org/search?author1=Akira+Yamada&sortspec=date&submit=Submit), [M. Shigemori](http://clincancerres.aacrjournals.org/search?author1=Minoru+Shigemori&sortspec=date&submit=Submit), [K. Itoh](http://clincancerres.aacrjournals.org/search?author1=Kyogo+Itoh&sortspec=date&submit=Submit) and [R. Tanaka](http://clincancerres.aacrjournals.org/search?author1=Ryuichi+Tanaka&sortspec=date&submit=Submit). Immunologic Evaluation of Personalized Peptide Vaccination for Patients with Advanced Malignant Glioma. Clin Cancer Res August 15, 2005 11; 5900. CD8+ T cells release IFN-γ in response to pancreatic tumor cells: [M. Ito](http://cancerres.aacrjournals.org/search?author1=Masaaki+Ito&sortspec=date&submit=Submit), [S. Shichijo](http://cancerres.aacrjournals.org/search?author1=Shigeki+Shichijo&sortspec=date&submit=Submit), [N. Tsuda](http://cancerres.aacrjournals.org/search?author1=Naotake+Tsuda&sortspec=date&submit=Submit), [M. Ochi](http://cancerres.aacrjournals.org/search?author1=Mika+Ochi&sortspec=date&submit=Submit), [N. Harashima](http://cancerres.aacrjournals.org/search?author1=Nanae+Harashima&sortspec=date&submit=Submit), [N. Saito](http://cancerres.aacrjournals.org/search?author1=Norio+Saito&sortspec=date&submit=Submit), and [K. Itoh](http://cancerres.aacrjournals.org/search?author1=Kyogo+Itoh&sortspec=date&submit=Submit). Molecular Basis of T Cell-mediated Recognition of Pancreatic Cancer Cells. Cancer Res March 3, 2001 61; 2038-46.  CD4+ T cells can be generated in vivo towards lung cancer patients. M. Harada, R. Gohara, S. Matsueda, A. Muto, T. Oda, Y. Iwamoto, and K. Itoh. In Vivo Evidence That Peptide Vaccination Can Induce HLA-DR-Restricted CD4^+^ T Cells Reactive to a Class I Tumor Peptide. Journal of Immunology, 2004, 172: 2659-2667. |
| ***WHSC2*** | *Wolf-Hirschhorn Syndrome Candidate 2* | Wolf-Hirschhorn syndrome candidate 2 (Whsc2) Whsc2 mRNA was made annd CTLs could be developed that responded towards glioma cells: [N. Yajima](http://clincancerres.aacrjournals.org/search?author1=Naoki+Yajima&sortspec=date&submit=Submit), [R. Yamanaka](http://clincancerres.aacrjournals.org/search?author1=Ryuya+Yamanaka&sortspec=date&submit=Submit), [T. Mine](http://clincancerres.aacrjournals.org/search?author1=Takashi+Mine&sortspec=date&submit=Submit), [N. Tsuchiya](http://clincancerres.aacrjournals.org/search?author1=Naoto+Tsuchiya&sortspec=date&submit=Submit), [J. Homma](http://clincancerres.aacrjournals.org/search?author1=Jumpei+Homma&sortspec=date&submit=Submit), [M. Sano](http://clincancerres.aacrjournals.org/search?author1=Masakazu+Sano&sortspec=date&submit=Submit), [T. Kuramoto](http://clincancerres.aacrjournals.org/search?author1=Terukazu+Kuramoto&sortspec=date&submit=Submit), [Y. Obata](http://clincancerres.aacrjournals.org/search?author1=Yayoi+Obata&sortspec=date&submit=Submit), [N. Komatsu](http://clincancerres.aacrjournals.org/search?author1=Nobukazu+Komatsu&sortspec=date&submit=Submit), [Y. Arima](http://clincancerres.aacrjournals.org/search?author1=Yoshimi+Arima&sortspec=date&submit=Submit), [A.Yamada](http://clincancerres.aacrjournals.org/search?author1=Akira+Yamada&sortspec=date&submit=Submit), [M. Shigemori](http://clincancerres.aacrjournals.org/search?author1=Minoru+Shigemori&sortspec=date&submit=Submit), [K. Itoh](http://clincancerres.aacrjournals.org/search?author1=Kyogo+Itoh&sortspec=date&submit=Submit). and [R. Tanaka](http://clincancerres.aacrjournals.org/search?author1=Ryuichi+Tanaka&sortspec=date&submit=Submit). 2005. Immunologic evaluation of personalized peptide vaccination for patients with advanced malignant glioma. Clin Cancer Res: 11:5900-5911. CD8+ T cells release IFN-γ in response to pancreatic tumor cells: [M. Ito](http://cancerres.aacrjournals.org/search?author1=Masaaki+Ito&sortspec=date&submit=Submit), [S. Shichijo](http://cancerres.aacrjournals.org/search?author1=Shigeki+Shichijo&sortspec=date&submit=Submit), [N. Tsuda](http://cancerres.aacrjournals.org/search?author1=Naotake+Tsuda&sortspec=date&submit=Submit), [M. Ochi](http://cancerres.aacrjournals.org/search?author1=Mika+Ochi&sortspec=date&submit=Submit), [N. Harashima](http://cancerres.aacrjournals.org/search?author1=Nanae+Harashima&sortspec=date&submit=Submit), [N. Saito](http://cancerres.aacrjournals.org/search?author1=Norio+Saito&sortspec=date&submit=Submit), and [K. Itoh](http://cancerres.aacrjournals.org/search?author1=Kyogo+Itoh&sortspec=date&submit=Submit). Molecular Basis of T Cell-mediated Recognition of Pancreatic Cancer Cells. Cancer Res March 3, 2001 61; 2038-46.Surgically resected adult and pediatric tissue can express this antigen. Zhang JG, E. Newcomb, D. Zagzag, C. Kruse, L. Driggers, N. Hoa, M.R. Jadus. 2008. Tumor antigenic profiles of adult and pediatric brain tumors. J. Neuro-Oncol. 88:65-76.By proteomic studies and confirmed by western blotting Wshc2 was found to be heavily expressed within gliomas, but not normal tissue: Li, J., Yin, C., Okamoto, H, Mushlin, H, Balgley, BM, Lee, CS, Yuan, K, Ikejiri, B, Glasker, S, Vortmeyer, AO, Oldfield, EH, Weil, R.J., Zhuang, Z. [Identification of a novel proliferation-related protein, WHSC1 4a, in human gliomas.](http://www.wikigenes.org/e/ref/e/18182627.html) Neuro-oncology 10:45-51 (2008) Whsc2 CTLs lysed GBM cell lines: Y. Niu, Y. Terasaki, N. Komatsu, M. Noguchi, S. Shichijo, K. Itoh. Identification of peptides applicable as vaccines for HLA-A26-positive cancer patients. Cancer Science [100](http://onlinelibrary.wiley.com/doi/10.1111/cas.2009.100.issue-11/issuetoc): 2167–2174, 2009. |
| ***YKL-40*** *(CHI3L1)* | *Chitinase 3-like 1* | **YKL-40 is a Glycosylated** Chondrocyte protein YKL-40 **F. Okano, A. Yoshizawa, M. Sato. and Hideho Okada.2006.** Identification of an HLA-A2-restricted CTL epitope in glioma associated antigen YKL-40. Proc. Amer. Assoc. Cancer Res. 47: **Abstract #2232. They showed CTLs could be generated against YKL-40 peptides in the context of HLA-A2. In this group’s follow-up clinical study: (H. Okada, P. Kalinski, R. Ueda, G. Kohanbash, et al. 2010. Induction of CD8+ T-cell responses against novel glioma-associated antigen peptides and clinical activity by vaccination with a-Type 1 polarized dendritic cells and polyinosinic-polycytidylic acid stabilized by lysine and carboxymethyl-cellulose in patients with recurrent malignant glioma. J. Clin. Oncol. 29:330-336.), lymphocytes responding to antigen-pulsed DC produced CD8+ cells that responded by ELISPOT towards YKL-40 peptide.** Surgically resected adult and pediatric tissue can express this antigen. Zhang JG, E. Newcomb, D. Zagzag, C. Kruse, L. Driggers, N. Hoa, M.R. Jadus. 2008. Tumor antigenic profiles of adult and pediatric brain tumors. J. Neuro-Oncol. 88:65-76. CTLs were generated against YKL-40 and killed GBM cell lines. Hoa N., L. Ge, Y. Kuznetsov, A. McPherson, A.N. Cornforth, J.T.H. Pham, M.P. Myers, N. Ahmed, V.S. Salsman, L.S. Lamb, Jr., J.E. Bowersock, Y.Hu, Y.H. Zhou, and M.R. Jadus. Glioma cells display complex cell surface topographies that resist the actions of cytolytic effector lymphocytes. Journal of Immunology 185:4793-4803, 2010. |
