# Supplementary material for: Differential Glioma-Associated Tumor Antigen Expression Profiles of Human Glioma Cells Grown in Hypoxia
Source: PLoS One. 2012 Sep 5;7(9):e42661. doi: 10.1371/journal.pone.0042661 (PMC3434178; doi:10.1371/journal.pone.0042661)
Supplement: Table S2 — qRT - PCR primers used for this study. (DOCX) [file pone.0042661.s002.docx]

| **Table S2:** qRT-PCR primers used for this study | |
| --- | --- |
| **Aim-2** | Forward: 5'-GCCTCACGTGTGTTAGATGC--3' |
|  | Reverse: 5'-ATCTTCGGGGTTTCACCAGC-3' |
| **Art-1** | Forward: 5'-TTGATGACCAGTACGCTGG-3' |
|  | Reverse: 5'-TCTGCATACACCTGGTTGG-3' |
| **Art-4** | Forward: 5'-CCTGTACAGTACTTCTCC-3' |
|  | Reverse: 5'-CAACCAGTCTCCTCTTG-3' |
| **B1-cyclin** | Forward: 5'-GCCACGAACAGGCCAATAAG-3' |
|  | Reverse: 5'-AAGGACCTACACCCAGCAGA-3' |
| **EphA2** | Forward: 5'-GCCTTCTTTAGACCCTCG-3' |
|  | Reverse: 5'-CCTCAACACAACCAAGCATC-3' |
| **Ezh2** | Forward: 5'-CAGGAACCTCGAGTACTG-3' |
|  | Reverse: 5’-CAGCTGGTGAGAAGGCAA-3' |
| **Fosl1** | Forward: 5'-CCAGAGACTTTGTAGATCC-3’ |
|  | Reverse: 5'- GTGGATCACAGGAAGAG-3' |
| **gp100** | Forward: 5'-ACAGGCCAACTGCAGAGG-3' |
|  | Reverse: 5'-CAGTTGGCGCCTGACCAG-3' |
| **Gnt-V** | Forward: 5'-GTGGTTGAGCAGCACCTG-3' |
|  | Reverse: 5’-CCAGAAGCTATCCACTTTGCC-3' |
| **Her2/neu** | Forward: 5'-CGACCCATTCAGAGACTG-3' |
|  | Reverse: 5'-GCACTCTGTACAAAGCCTG-3' |
| **HNRPL** | Forward: 5'-CTGCACTTCTTCAACGCC-3' |
|  | Reverse: 5'-CACAGAAGATGGCCGCTT-3' |
| **IL-13Rα2** | Forward: 5'-GGGACCTATTCCAGCAAGG-3' |
|  | Reverse: 5'-CAACTGTAGCAGTCACCAAGG-3' |
| **Mage-A1** | Forward: 5'-GCTGGAGAGTGTCATCAA-3' |
|  | Reverse: 5'-CTTCCTTCACGTCAATGC-3' |
| **MRP-3** | Forward: 5'-CCTGAGATTTCCTCCTGG- 3' |
|  | Reverse: 5'-CCAGTGTTTGCTATCAAGTCC-3' |
| **PTH-rP** | Forward: 5'-CCAAGGACATATTGCAGG-3' |
|  | Reverse: 5'-GCAGTTTCATAGAGCAATGG-3' |
| **PRAME** | Forward: 5-’CTGGAAGCTACCCACCTT-3' |
|  | Reverse: 5'-GGAGAGGAGGAGTCTAC-3' |
| **Sart-1** | Forward: 5'-GACAAGTACAGCCGGAGG-3' |
|  | Reverse: 5'-CCGTCTCATCCACGTATTCG -3' |
| **Sart-2** | Forward: 5'-GATTAGACAGAAAGCTCAGATT-3' |
|  | Reverse: 5'-CATCTGCAAAATCTAAAAGGTC-3' |
| **Sart-3** | Forward: 5'-CACATGTGCCCGTCATTC-3' |
|  | Reverse: 5'-GCTGACTTCCCTCAGAC-3' |
| **Sox 11** | Forward: 5'-GTAGTGGTGATGATGATGATG-3' |
|  | Reverse: 5'-GCGTCACGACATCTTATC-3' |
| **Survivin** | Forward: 5'-GGGAGGAAGAAGGCAGTG-3' |
|  | Reverse: 5'-GCCTCAACAACATGAGGTCC-3' |
| **hTert** | Forward: 5'-GATGAGTGTGTACGTCG-3' |
|  | Reverse: 5'-CAACTTGCTCCAGACAC-3' |
| **Trp-1** | Forward: 5'-CACAAAACCACCTGGTTGAA-3' |
|  | Reverse: 5'-CCAGCTTTGAAAAGTATGCC-3' |
| **Trp-2** | Forward: 5'-CCTGTCTCTCCAGAAGTTTG-3' |
|  | Reverse: 5'-CAGAGTCCCATCTGCTTTATC-3' |
| **Ube2V1** | Forward: 5'-GTGCCCAAGGGGTTTTAGGA-3' |
|  | Reverse: 5'-GTGCCCAAGGGGTTTTAGGA-3' |
| **Whsc2** | Forward: 5'-CATTTCTCTGGGGTATTTGG-3' |
|  | Reverse: 5'-CGTTGAGTCCAAAAAGTGTC-3' |
| **YKL-40** | Forward: 5'-GGACACCATTTTGGCAAG-3' |
|  | Reverse: 5'-CCTAAGTGAAGGTTTCAAGC-3 |
