# Supplementary figures and images for: Differential Glioma-Associated Tumor Antigen Expression Profiles of Human Glioma Cells Grown in Hypoxia
Source: PLoS One. 2012 Sep 5;7(9):e42661. doi: 10.1371/journal.pone.0042661 (PMC3434178; doi:10.1371/journal.pone.0042661)

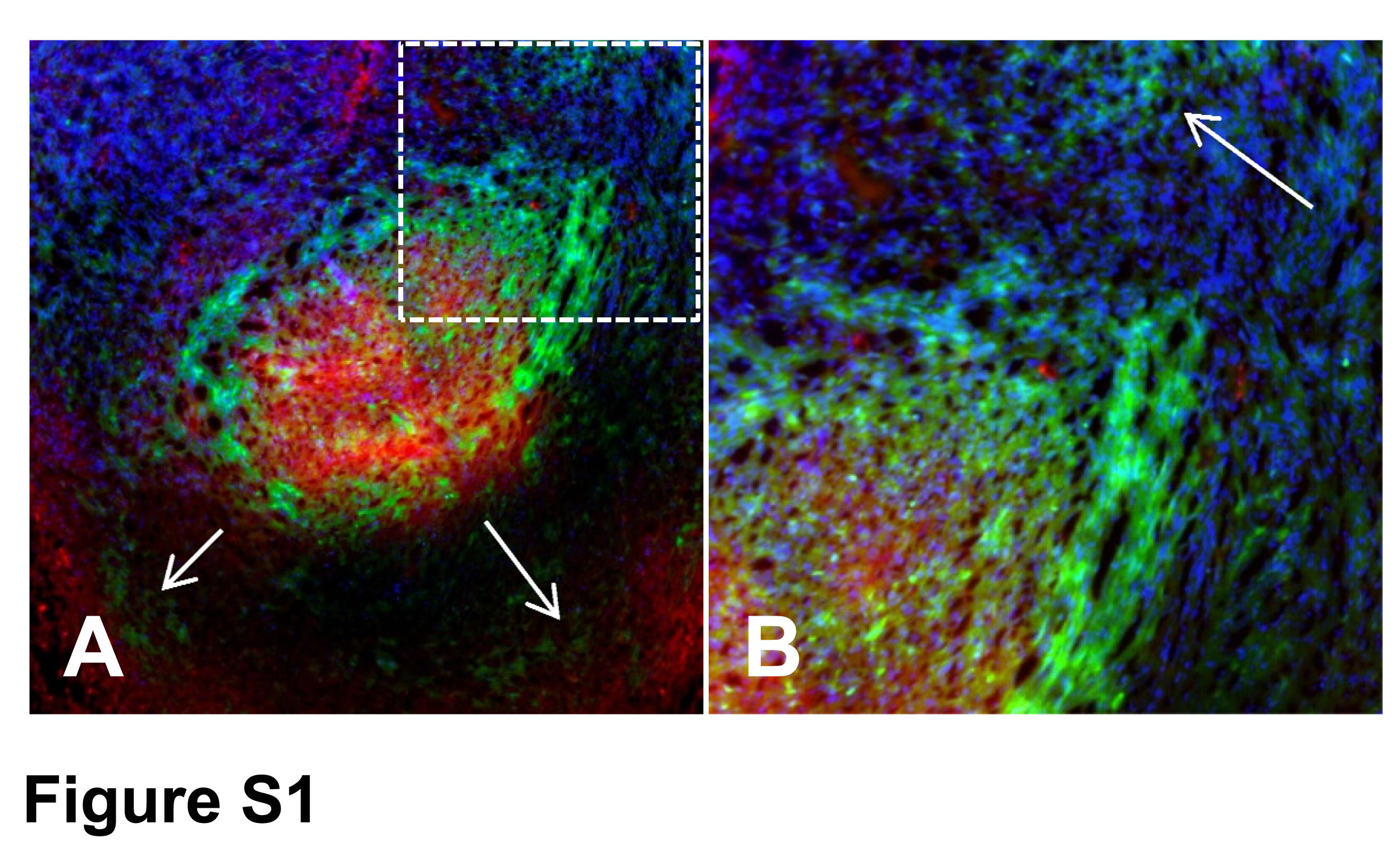

Supplement: Figure S1 — Two-color fluorescent microscopy shows co-localization of Whsc2 and YKL-40 with HIF-1α in U251 CD133+ “stem-like” cell induced tumors. Human U251 neurosphere “stem cells” were implanted into the brain of a nude mouse. When the glioma was showing an effect on the mouse, the mouse was euthanized and the brain was removed. Serial sections were cut and then stained with either anti-HIF-1α (red), Whsc2 (green) or YKL-40 (green). The nuclei are stained with DAPI (blue). Panel B shows a magnified region that was highlighted from Panel A. (TIF) [file pone.0042661.s003.tif]
